# Unveiling Marine Vesicle Uptake in *Vibrio* spp: Taxonomic and Environmental Insights

**DOI:** 10.1002/mbo3.70311

**Published:** 2026-05-10

**Authors:** Nadefa Adda Nekrouf, Lucia Maestre‐Carballa, Monica Lluesma‐Gomez, Esther Rubio‐Portillo, Manuel Martinez‐Garcia

**Affiliations:** ^1^ Department of Physiology, Genetics, and Microbiology University of Alicante, Carretera San Vicente del Raspeig, San Vicente del Raspeig Alicante Spain; ^2^ Multidisciplinary Institute for Environmental Studies Ramon Margaleff (IMEM) University of Alicante, Carretera San Vicente del Raspeig, San Vicente del Raspeig Alicante Spain; ^3^ University Mustapha Stambouli Mascara Algeria

**Keywords:** corals, extracellular vesicle, *interactions*, marine, pathogen, pathogenesis, vesicle, *Vibrio*, *Vibrio coralliilyticus*, *Vibrio kanaloae*

## Abstract

Extracellular vesicles (EVs) are involved in diverse functions in nature, from biogeochemical cycles to pathogenesis. Here, we investigate whether taxonomy represents a boundary that constrains the uptake of vesicles between different marine species. We focus on *Vibrio* spp. since they are ubiquitous and play different roles, including in diseases affecting humans and marine organisms, such as corals. Spectral flow cytometry data showed intra‐ and inter‐species uptake of EVs from the pathogenic *Vibrio kanaloae* and *Vibrio coralliilyticus* by different *Vibrio* strains and species, including other marine and non‐marine Gram‐negative and Gram‐positive recipient bacteria. EV fusion efficiencies with recipient cells ranged from 53% to 99%, although these values may vary depending on the nutrient status of the cells. Data suggest that EV fusion between cells from different taxa can occur regardless the phylogenetic distance between the EV donor and recipient cell and the quantity of EVs added. Data revealed that nutrient conditions did not play a significant role in the number of EVs released by the analyzed *Vibrio* spp., although differences were observed in the amount of DNA exported in EVs (*p*‐value = 0.02), with more bulk vesicular DNA cargo in diluted nutrient conditions. Nearly the entire genome of the targeted *Vibrio* spp. was detected in the EV fraction. Our data suggest that taxonomic distance between the EV donor and recipient cell is not a major boundary.

## Graphical abstract

Extracellular vesicles are involved in diverse functions in nature from microbial interactions to pathogenesis. Here, we investigate whether taxonomy represents a boundary that constrains the uptake of vesicles between different marine bacteria including ubiquitous Vibrio spp. involved in marine organism diseases, such as corals. Our data suggest that taxonomic distance between vesicle donor and recipient cell is not a major boundary.

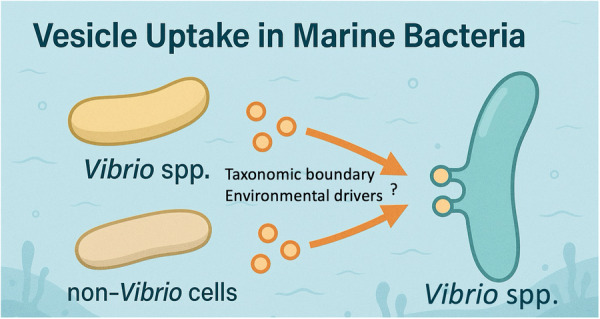

## 1 Introduction

Extracellular vesicles (EVs), which are secretory nanoparticles, are ubiquitously produced by most bacteria, demonstrating diverse biological functionalities in nature and broad applications in different fields, such as immunology and biotechnology (Nagakubo et al. 2020; Bose et al. 2020). The ubiquity of EV production across all life domains hints at their capacity to convey highly specific signals that modulate cell behavior, physiology, morphology, and susceptibility even to viral entities (Schatz and Vardi 2018).

Different terms are employed across organisms to refer to EVs, such as outer membrane vesicles (OMVs) in Gram‐negative bacteria and membrane vesicles (MVs) in Gram‐positive bacteria. To maintain uniformity, the International Society for EVs (ISEV) recommends the term EV as a comprehensive descriptor for particles naturally released from cells, delimited by a lipid bilayer and incapable of replication (Bose et al. 2020; Théry et al. 2018).

EVs have been obtained from bacteria inhabiting diverse environments, including freshwater and saline habitats, biofilm structures, soils, intracellular spaces within eukaryotic cells, and mammalian host organisms, including human bacterial pathogens (Bose et al. 2020; Biller et al. 2014; Schooling and Beveridge 2006; Beveridge 1999; Beveridge et al. 1997; Brandtzaeg et al. 1992; Hellman et al. 2000; Schwechheimer and Kuehn 2015). They have been found to contain various biomolecules, including proteins, nucleic acids, phospholipids, adhesins, and lipopolysaccharides, that overall play a vital role in processes such as virulence, nutritional sensing, competition, and cell‐to‐cell communication (Ahmed and McKay 2024).

In aquatic habitats, recent research has unveiled a substantial presence of EVs in marine environments, reaching up to 10⁶ EVs/mL and originating from diverse taxa (Biller et al. 2014; Lücking et al. 2023). Vesicle production has been documented in several highly prevalent marine heterotrophs and autotrophs, including *Marinobacter* and *Prochlorococcus*, which are commonly used as model systems for studying EV production (Lücking et al. 2023; Linney et al. 2022; Domingues and Nielsen 2017). These EVs constitute a previously undetected component of the dissolved organic fraction in marine ecosystems (Biller et al. 2014). Additionally, they hold the potential to serve as pivotal carriers for genetic and biogeochemical processes, introducing a formerly overlooked dimension into the intricate dynamics of marine systems (Biller et al. 2014).

Among the different relevant marine microbes, *Vibrio* are heterotrophic bacteria widely distributed in marine environments across the globe. Numerous *Vibrio* species can be found throughout aquatic ecosystems and are known for their exceptionally rapid growth, enabling them to thrive and prevail, especially in eutrophic environments (Aiyar et al. 2002; Macián et al. 2000; Thompson et al. 2003). These species display versatility in their growth patterns, thriving as free‐living organisms, forming associations, and engaging in mutualistic or symbiotic relationships with various aquatic organisms (Macián et al. 2000; Thompson et al. 2003). Furthermore, *Vibrio* spp. are frequently linked to diseases in humans and marine organisms, including shellfish, fish, and corals (Mitchell and Mitchell 2010; Rubio‐Portillo et al. 2018). The occurrence of *Vibrio* species is strongly influenced by temperature, making them more prevalent in warm waters and temperate regions, where their abundance tends to rise during warmer seasons (Rubio‐Portillo et al. 2018; Wright et al. 1996; Vezzulli et al. 2012). Consequently, global warming may facilitate their proliferation and increase the risk of disease outbreaks caused by *Vibrio* pathogens (Harvell et al. 2002; Baker‐Austin et al. 2013; Vezzulli et al. 2013, 2010).

Several *Vibrio* species, notably *Vibrio coralliilyticus* and *Vibrio kanaloae*, are known to infect corals and clams (Thompson et al. 2003; Huang et al. 2021; Rubio‐Portillo et al. 2020), respectively. These species produce EVs that are believed to play a role in the regulation of the coral microbiome and have important implications for coral health and disease in the face of climate change (Li et al. 2024). In the case of other pathogenic *Vibrio* spp., EVs play a major role in delivering toxins (Zingl et al. 2021). Indeed, in other models, such as *Vibrio fischeri*, EVs are involved in the development of the host (Aschtgen et al. 2015). Therefore, in this study, we aim to explore the extent to which EVs from different *Vibrio* species interact with other marine bacteria and whether phylogenetic distance acts as a boundary. Furthermore, we characterize the DNA content and environmental drivers of vesicle production, providing deeper insights into the EV dynamics of *Vibrio* spp., which play a major ecological role in marine ecosystems.

## 2 Material and Methods

### 2.1 Bacterial Cultures, Isolation, and Identification

*Escherichia coli* CECT 101, *Bacillus cereus* CECT 193, and *Staphylococcus aureus* CECT 435 were grown in LB at 30°C for 48 h. These strains were provided by the Spanish Type Culture Collection (Valencia, Spain). All *Vibrio* strains used in this study, except *Vibrio kanaloae*, were kindly provided by Dr. Esther Rubio‐Portillo (University of Alicante; Supporting Information S1: Table S1) and were grown in LB supplemented with 3% NaCl at 30°C for 48 h. *Vibrio coralliilyticus* was originally isolated from the mucus tissue of the coral *Oculina patagonica* collected from the Tabarca island (June 2011, Mediterranean Sea) (Rubio‐Portillo et al. 2020, 2014). *Arcobacter roscoffensis* and *Vibrio kanaloae* were isolated in pure culture from a seawater sample collected on May 31, 2022, from Cape Huertas (Alicante, Spain). For that, 5 μL of seawater was inoculated onto Difco Marine Agar 2216 plates at 30°C overnight. To ensure the purity of the culture, single colonies were picked and streaked onto fresh culture medium five consecutive times. Identification of isolated colonies was performed by sequencing 16S rRNA gene as follows. In brief, biomass from isolated colonies was suspended in 200 μL of sterile buffer TE and boiled at 100°C for 5 min. Then, sample was centrifuged at 13,000 x g for 10 min at 4°C, and supernatant containing DNA was measured using the Qubit HS dsDNA assay kit from Thermo Fisher Scientific (Reference: Q32854) and 16S rRNA gene was PCR amplified using the following conditions: 7.5 ng of extracted DNA, 2.5 μL of 10x PCR reaction buffer, 0.75 μL of 50 mM MgCl2, 0.5 μL of 10 mM dNTPs, and 1 μL of 10 μM for each primer 27 F (5′‐AGR GTT YGA TYM TGG CTC AG‐3′) and 907 R (5′‐CCG TCA ATT CMT TTR AGT TT‐3′), 0.1 μL of Taq polymerase (Thermo Fisher Scientific, Ref: 10342‐020), and sterile mQ water was added to reach a final volume of 25 μL. PCR cycling conditions were as follows: an initial denaturation at 94°C for 2 min, followed by 35 cycles of denaturation at 94°C for 30 s, annealing at 55°C for 30 s, and extension at 72°C for 1 min. A final extension was performed at 72°C for 5 min. PCR products were subsequently purified using the GeneJET PCR Purification Kit from Thermo Scientific and sequenced using the Sanger sequencing technique with the same 27 F and 907 R primers in the Sequencing Center of the University of Alicante. Taxonomic identification was performed using the SILVA SINA online aligner (Pruesse et al. 2012) (https://www.arb-silva.de/aligner/) against the SILVA SSU rRNA database (release v139). Sequences were aligned to reference sequences, and taxonomy was assigned using a least common ancestor (LCA) approach based on sequence similarity.

After incubation, bacterial cells used for downstream analyses, such as for spectral flow cytometry, were harvested by centrifugation at 6000 x g at 4°C for 20 min. The cell pellets were resuspended in HEPES (Sigma, Ref.H3375) 10 mM, pH 7.4 containing 0.85% NaCl previously filtered through a 0.02 μm Whatman Anotop filter (Ref. 6809‐1002 or 6809‐4002).

### 2.2 EVs Isolation and Visualization

Both *Vibrio coralliilyticus* (strain Vic‐Oc‐068; CECT 30097) and *Vibrio kanaloae* were grown as described above, and the supernatant containing EVs was processed as follows. Between 70 and 165 mL of volume from *Vibrio coralliilyticus* and *Vibrio kanaloae* was centrifuged at 6000 x g for 20 min at 4°C to remove cells. This step was repeated twice to ensure the removal of bacterial cells. To prevent contamination with bacteria, the supernatant was filtered twice suquentially through 0.22 μm PES filter membrane (LLG‐Syringe filters SPHEROS, ref: 220329‐322‐A). Then, the 0.22 μm filtered supernatants underwent a concentration step using 15 mL Sartorius Vivaspin 20 Centrifugal Concentrators (Fisher Scientific, ref: VS2042) or Amicon Ultra 15 mL (100 KDa; Merck Millipore; Ref. UFC910024) up to a volume of 0.5–1 mL. EVs were then isolated using OptiPrep density gradient (Sigma, Ref. D.1556) as described (Pérez‐Cruz et al. 2013). For this, concentrated samples underwent ultracentrifugation (Beckman L‐70 Ultracentrifuge; swinging‐bucket rotor SW40Ti) at 186,000 x g overnight at 4°C. Density fractions 20, 25, and 30% of OptiPrep were further washed for an hour at 100,000 x g with 10 mM buffer HEPES, 0.85% NaCl, adjusted to a pH of 7.4, previously filtered through 0.02 μm Whatman Anotop filter (Ref. 6809‐1002 or 6809‐4002). The resulting pellet containing EVs from each OptiPrep gradient and sample was then carefully resuspended in 500 μL of the above described HEPES buffer, and aliquots were frozen at ‐80°C until further use. To confirm the proper isolation of EVs, 5 μL of each fraction was analyzed using TEM, following the procedure described by Maestre‐Carballa et al (Maestre‐Carballa et al. 2019), and FESEM, as outlined by Noble et al (Noble et al. 2020).

### 2.3 EVs Fluorescent Labeling

500 μL of EVs of *V. coralliilyticus* strain Vic‐Oc‐068 were dyed with 12.5 μL of the fluorophore FM4‐64 (100 μg/mL; Invitrogen, Thermo Fisher Scientific, Ref. T13320) incubated for 5 min at room temperature in dark conditions. The volume was adjusted to 700 μL using HEPES buffer (containing 0.85% NaCl) and analyzed using NanoSight LM10HS instrument (Malvern, UK) equipped with fluorescence and dispersion modes, in the proteomics facilities of the CIPF (Valencia, Spain). HEPES buffer (10 mM, 0.85% NaCl, pH 7.4) was used as a blank and processed as the sample. Five replicated videos were obtained for each sample.

250 μL of *Vibrio kanaloae* cells, 250 μL of *V. kanaloae* EVs (Optiprep fractions 20, 25% and 30%), and 250 μL of 0.02 μm‐filtered HEPES (10 mM, 0.85% NaCl, pH 7.4) were stained with 4 μL of FM4‐64, incubated for 1 h at room temperature in the dark and washed two times using an Amicon Ultra 15 mL (100 KDa; Merck Millipore; Ref. UFC910024) and 5 mL of 0.02 μm‐filtered HEPES buffer each wash (2500 x g, 2.5 min). A negative control, consisting of undyed cells was also included. Each measurement was performed in duplicate. To evaluate the efficiency of the washing steps for FM4‐64 using AMICON, before the incubation and after each wash, the fluorescence intensity (505‐15/730‐20) was measured with CLARIOstar plate reader (BMG LABTECH, Germany).

For the fusion experiment two different fluorophores were used to dye the EVs to increase the signal: Alexa fluor 488 5‐SDP or AF‐488 (1 mg/mL; DMSO; Thermo Fisher Scientific; Ref. A30052) which targets proteins, and SynaptoGreen C4 or FM1‐43 (5 mg/mL; DMSO; Biotium; Ref. 70020), which targets membranes, were incubated at 37°C for 10 min with gentle agitation and darkness before their use.

A total of 100 μL of each fraction (20%, 25%, and 30%) were thawed at 4°C, then mixed and vortexed. Simultaneously, 300 μL of HEPES (10 mM, 0.85% NaCl, pH 7.4) filtered through 0.02 μm was prepared as a negative control and processed identically to the sample. AF‐488 (0.3 μL) and FM1‐43 (0.3 μL) were added to both the sample and the negative control. After vortexing, they were incubated at 30°C in darkness at 400 rpm for 1 h. Excess of dye was removed washing by 3 times with 5 mL of 0.02 μm‐filtered HEPES using Amicon Ultra 15 mL (100 KDa; Merck Millipore; Ref. UFC910024) and centrifuging at 2500 x g for 2.5 min. After each wash, the concentrate was resuspended using a 100 μL pipette to ensure that vesicles and fluorophores were properly detached from the Amicon membrane. The final volumes of the sample and the negative control were adjusted to 250 µL with 0.02 µm‐filtered HEPES buffer.

CLARIOstar plate reader (BMG LABTECH, Germany) was used to confirm that three consecutive washes with HEPES buffer and an Amicon membrane, effectively removed most of the free dye (AF‐488 and FM1‐43; 428.5‐9/570‐100) present in vesicles.

### 2.4 Statistical Analysis and Fluorescence Assessment

Fluorescence labeling was assessed by comparing unstained EVs, bacterial cells, and HEPES buffer baseline controls with EVs and HEPES after staining, as well as after staining followed by washing. Statistical comparisons were used to evaluate fluorescence differences between independently processed aliquots within each experiment. For each condition, fluorescence was measured in three experimental replicates, and each replicate was measured in technical triplicate. Technical replicates were averaged prior to statistical analysis, so that each tube contributed a single value. Measurements reaching the upper detection limit of the instrument (≥ 260,000 RFU) were considered saturated and were excluded from quantitative statistical comparisons. Differences between EVs and HEPES were assessed only for datasets acquired within the linear detection range of the instrument. Because fluorescence values were analyzed on a multiplicative scale, statistical comparisons were performed using Welch's t‐test on log10‐transformed RFU values

To test the efficacy of the fluorophores combination AF‐488 and FM1‐43 in labeling EVs from natural marine samples, we collected 25 L of sea‐water (06/06/23) at Cape Huertas in the Mediterranean Sea (Alicante, Spain; 38°21′38″ N, 0°25′22″ W). The sample was processed as indicated (Nekrouf et al. 2025). Subsequently, 300 μL of EVs isolated from the Optiprep fractions 20, 25% and 30% were stained following the aforementioned protocol and examined with confocal microscopy.

### 2.5 Bacterial Uptake and Fusion of Purified *Vibrio* EVs

A total of 100 μL of cells (OD_600_ = 0.3–0.4) from seven different species including marine and non‐marine bacteria (Table S1) were mixed with 200 μL of fluorescently labeled and washed *Vibrio* EVs produced by *V. kanaloae* strain 1 C or *V. coralliilyticus* strain Vic‐Oc‐068. Then, they were incubated in dark conditions for 0.5–1 h at 250 rpm and 30°C. Controls in the experiment were comprised of 200 μL of dyed and washed HEPES buffer as described above and incubated as before. For the observation of the EV uptake and fusion by different bacterial cultures, confocal and super‐resolution microscopy, and spectral flow cytometry were used as described below.

### 2.6 Spectral Flow Cytometry

Aurora Spectral Cytometer from Cytek (Research Technical Services, University of Alicante) equipped with three excitation lasers (405 nm, 488 nm and, 640 nm) and 38 fluorescent parameter detectors (16‐Violet, 14‐Blue and 8‐Red). The analysis was performed at a low flow rate for 30 s using SpectroFlo software. The detector gains were set as follows: FSC at 500, SSC at 300, and SSC‐B at 368 (Research Technical Services of the University of Alicante, Spain). In addition to the fusion experiments, undyed cells, dyed and washed vesicles, and both dyed and undyed HEPES were analyzed as controls to validate our results. Unless otherwise indicated, serial dilutions using 0.02 μm filtered HEPES were performed until the EVs‐labeled cells population (from the sample: Cells+EVs) contained approximately 9000 events. The same dilution of HEPES and cells was then analyzed as a control. After the initial analysis, samples containing vesicles and cells were filtered through either a 0.2 µm or 0.02 µm filter to remove cells or both vesicles and cells selectively. The filtered samples were then reanalyzed by flow cytometry. EVs‐fused cells gate was established by comparing the sample (Cells + EVs) with the negative control (Cells + HEPES), which was considered as the effect of the fluorophores on their own. The percentage of cells fusing with vesicles was estimated by comparing, for each sample, the number of events within the EVs‐labeled cell gate (BH4 vs. SSCH) to the number of events found in the all‐cells gate (FSCH vs. SSCH) for the same sample. Background subtraction was conducted by subtracting events in the all‐cells gate of the plot FSCH versus SSCH present in the HEPES labeled and washed negative control. Background subtraction was also applied to the EVs‐labeled cell gate. EVs‐to‐cell ratio was calculated by dividing the number of events in the EVs gate from two serial dilutions, where the events corresponded to the dilution factor, by the number of cells in the all‐cells gate (FSCH vs. SSCH). All samples were analyzed using a low flow rate for 30 s, which was equivalent to analyze ~4.6 μL per sample. Background subtraction was performed in both cases. The Average Nucleotide Identity (ANI) for the strains of *V. coralliilyticus* was calculated using JSpeciesWS Online Service (Richter et al. 2016), with *V. coralliilyticus* strain Vic‐Oc‐068 as the reference. These values were then compared with the percentage of EV‐s fused cells for each *V. coralliilyticus* used, using a regression analysis.

Because these measurements were obtained from within‐experiment comparisons rather than from fully independent biological replicates across recipient taxa, the fusion/association data were interpreted as descriptive evidence of consistent patterns among taxa rather than as a fully replicated inferential comparison.

### 2.7 Confocal and Super‐Resolution Microscopy of EVs

Confocal microscopy samples were prepared by adding 8 μL of stained and washed EVs/HEPES or fusion samples on a high‐precision cover glass (Marienfeld, No. 1.5H, Ref. 0107032). The sample was spread and dried at room temperature for 30 min. The mounting media used was Citifluor AF1 (Electron Microscopy Sciences, glycerol‐based antifadent mounting solution, Ref. 17970‐100). The samples were mounted, sealed and, observed under a 100X oil objective. The microscope used was a Zeiss LSM 800 confocal microscope, equipped with Axio Imger Z.2, two GaAsP detectors of high sensitivity, and three excitation lines of laser diode: 405 nm (UV), 488 nm (blue), 561 nm (green) and 640 nm (red) from the Research Technical Services of the University of Alicante (Spain).

EVs from *V. coralliilyticus* strain Vic‐Oc‐068 stained with SynaptoGreen C4 (FM1‐43) and washed with HEPES buffer were loaded onto a high‐precision coverslip (Marienfeld, No. 1.5H, Ref. 0107032), which had been pre‐treated with plasma to promote sample adhesion. Fluoromount‐G (ThermoFisher Scientific, Ref. 00‐4958‐02) was used as mounting media. The samples were observed using a Zeiss LSM 980 super‐resolution microscope equipped with Airyscan 2 and lasers at 405, 488, 561, and 639 nm (Advanced Light Microscopy Unit, CRG, Barcelona, Spain). Image processing and analysis were performed using the open‐source software ImageJ.

### 2.8 EVs Quantification By Nanoparticle Tracking Analysis (NTS)

The methodology described in Biller et al (Biller et al. 2014). was used for EVs quantification. The concentrations of EVs were measured using a NanoSight NS300 instrument (Malvern Panalytical, Malvern, UK) equipped with a blue laser module (488 nm) and NTA software V3.2. at the Prince Felipe Research Center in Valencia, Spain. We followed the manufacturer's guidelines, diluting samples to achieve an average particle count per field ranging from 20 to 60. Recording of each sample involved five 30‐second videos, and thorough flushing of the sample chamber with 18.2 MΩ cm^−1^ water (Milli‐Q; Millipore) occurred between samples to prevent particle carryover. During the NTA measurement, particles below 200 nm were detected and counted in the dispersion mode of the instrument.

### 2.9 Microcosm Experiment of *Vibrio kanaloae* 1C

Difco Marine Broth 2216 medium was diluted for growing *Vibrio kanaloae* strain 1 C by mixing it with Milli‐Q (mQ) water at a ratio of 1/5, creating a fivefold dilution.

Six Erlenmeyer flasks of 190 mL of the Difco Marine Broth 2216 medium diluted to a final ratio 1:5 with sterile Milli‐Q (mQ) water were inoculated each with 10 mL of *Vibrio kanaloae* strain 1 C previously cultured in standard Difco Marine Broth 2216 medium. Following inoculation, three flasks were placed in an incubator (Lan Techniques) at 18°C with shaking at 150 rpm. Simultaneously, the other three flasks were transferred to dialysis tubing cellulose membranes (Sigma‐Aldrich, ref: D9652‐100FT, width: 33 mm, 14 KDa), immersed in an aquarium filled with natural seawater collected on December 9th, 2022, from Cape Huertas (Mediterranean Sea, Alicante), and incubated at room temperature (approximately 18°C–20°C). For light exposure, a lamp was used for 12 h. Both sets of cultures, the Erlenmeyer flasks, and the dialysis tubing cellulose membrane culture microcosms underwent a 1‐week incubation period. In addition, another set of three Erlenmeyer with *Vibrio kanaloae* strain 1 C cultured in standard (not diluted) Difco Marine Broth 2216 medium was grown and incubated overnight at 30°C with shaking at 150 rpm. Following the 1‐week incubation period, ≈165 mL of volume from replicates of each of three different culture conditions were used for EV isolation as described above in section EVs isolation from *Vibrio* spp. cultures, and then DNA was extracted and sequenced as detailed below.

### 2.10 Extraction of Vesicular DNA From *Vibrio* spp. and Sequencing

A total of ≈500 µL of purified EVs from *V. coralliilyticus* (20%, 25%, and 30% Optiprep gradients) or *V. kanaloae* obtained from the 20% Optiprep gradient were concentrated using Amicon Ultra 0.5 mL centrifugal filters (Merck Millipore Ltd., Ref. UFC510096) to a final volume of 200 μL. The supernatant was treated with DNase and RNase as indicated (Maestre‐Carballa et al. 2024), and DNA was subsequently extracted with QiAamp MinElute Virus Spin Kit (Qiagen, Ref. 57704) without using a carrier. The extracted DNA was quantified using the Qubit HS dsDNA assay kit by Thermo Fisher Scientific (Ref. Q32854) and stored frozen at −80°C until use. The Illumina DNA Prep Kit (IDT, Ref. 20026930) was employed following the manufacturer's protocol to generate libraries from extracted vesicular DNA that were sequenced using HiSeq or HiSeq X technology (150PE, 1 lane) at the Macrogen facility (Seoul, Republic of Korea). To obtain a metagenomic library, multiple displacement amplification (MDA) was performed on the *Vibrio coralliilyticus* EVs following the methodology described by De La Cruz Peña et al (De La Cruz Peña et al. 2018).

### 2.11 Bioinformatic Analysis of Sequenced Data of Vesicular DNA and Genome Sequencing of *Vibrio kanaloae* Strain 1C

Genome sequencing of *Vibrio kanaloae* strain 1 C was performed as follows. A total of 10 mL of pure culture was centrifuged 2 times for 20 min at 6000 x g at 4°C to pellet the cells. Then the pellet was resuspended in 500 μL of buffer TE. 250 μL was used to extract the genomic DNA using the DNeasy PowerSoil Pro Kit (QIAGEN). This extraction process ensured the separation of DNA from residual contaminants, including RNA, proteins, and lipids, employing a thorough RNAse, DNase_free kit (Sigma‐Aldrich, Reference 11119915001) following the manufacturer's protocol. Accurate quantification of DNA concentrations was achieved using the Qubit High Sensitivity dsDNA assay kit (Thermo Fisher Scientific, Reference Q32854*). Vibrio kanaloae* strain 1 C genome sequence was obtained using long‐read sequencing technology by High Fidelity Pacific Bioscience (HiFi PacBio) on the Sequel II system (PacBio, USA) following the manufacturer's protocol (Procedure Checklist: Preparing Whole‐Genome and Metagenome Libraries Using the SMRTbell Prep Kit 3.0, 102‐166‐600 REV02, PacBio) using the Sequel II Binding Kit 3.2 and the Sequel II Sequencing Kit 2.0 in the Genomics Center of FISABIO (Valencia, Spain). The genomics center delivered quality filtered reads according to HiFI Pacbio manufacturer's manual that were subsequently employed for genome assembly using the Flye assembler with default conditions (Kolmogorov et al. 2019). Gene prediction and annotation were performed with Prodigal using the Kbase bioinformatic platform (Arkin et al. 2018). *Vibrio* genome mapping against microbial metagenomes (Nekrouf et al. 2025) obtained from natural seawater collected at the same sampling point where this species was originally isolated was performed using Bowtie 2.0 (Langmead et al. 2009) with default parameters within the Geneious bioinformatics platform (Kearse et al. 2012).

The raw Illumina sequencing data from vesicular DNA were quality‐filtered using trimmomatic v0.36 (Bolger et al. 2014) with the following parameters: adapters/NexteraPE‐PE‐HiSeq. fa:2:30:10 LEADING:3 TRAILING:3 SLIDINGWINDOW:4:30 MINLEN:50. The quality of the filtered reads was assessed using FASTQC (https://github.com/s-andrews/FastQC). Quality‐filtered reads from EVs with sequence identities ≥ 70% were mapped to the *Vibrio coralliilyticus* strain Vic‐Oc‐068 genome or *Vibrio kanaloae* strain 1 C genome, with each EV sample mapped against its corresponding bacterial strain. This common nucleotide threshold has been used to consider only reads that displayed high nucleotide identity within the level of genus and species typically used in genomic and metagenomic surveys (Rodriguez‐R et al. 2021). The mapping results were visualized using the Enveomics R package (Rodriguez‐R and Konstantinidis 2016). The taxonomy of those reads whose identity compared with the *V. coralliilyticus* strain Vic‐Oc‐068 DNA was below 70% were compared with the nr_uk database using Kaiju (‐E 0.00001 ‐a greedy) (Menzel et al. 2016).

## 3 Results and Discussion

### 3.1 Fluorescence Labeling of EVs

EVs purified from natural seawater and bacterial cultures, such as *V. coralliilyticus* strain Vic‐Oc‐068 and *V. kanaloae* strain 1 C, were successfully labeled with the fluorophores AF‐488 and FM1‐43. Labeled EVs were subsequently detected using confocal microscopy, super‐resolution microscopy, and spectral flow cytometry (Supporting Information S1: Figures S1 and S2). TEM confirmed the presence of vesicles in the EV‐purified samples (Supporting Information S1: Figure S1). Negative controls included the same EV samples, filtered through a 0.02 μm filter to remove any biological nanoparticles. Additionally, the same sterile buffer HEPES used for vesicle suspension was also processed in parallel as a negative control, showing either no signal or significantly lower signal compared to the EV samples (Gate EVs from Supporting Information S1: Figure S1, panels C and D; Supporting Information S1: Figure S2, panel C; Supporting Information S1: Figure S3, panel D Welch Two Sample *t*‐test, *p*‐value = 2.561e‐06. Supporting Information S1: Figure S4). Previous studies have demonstrated the reliability of these fluorescent dyes for EV detection (Verstreken et al. 2008; Biller et al. 2022).

### 3.2 Potential Vesicle Uptake by Taxonomically Different Bacteria

EVs serve as a source of organic carbon and energy, supporting bacterial growth (Biller et al. 2014). Much of a vesicle's carbon energy content is found within the lipids, with each 100 nm diameter EV containing approximately 10,000 lipid molecules. Furthermore, the diverse proteins and metabolites exported within EVs represent potential labile organic carbon for co‐occurring bacteria in the same environment. Previous studies have shown that EVs can be uptaken by recipient cells, delivering their cargo, including DNA (Li et al. 1998; Tashiro et al. 2017; Tran and Boedicker 2017; Lee et al. 2022). Here, we used EVs purified from two marine *Vibrio* species—*V. coralliilyticus* strain Vic‐Oc‐068 and *V. kanaloae* strain 1 C—as a model. *Vibrio* spp. are common in coastal seawater and play important roles in environmental processes, such as the degradation of complex organic molecules, while stablishing various types of relationships with marine organisms, ranging from mutualism to coral pathogenesis (Macián et al. 2000; Thompson et al. 2003). The presence of these *Vibrio* strains in the natural seawater used for isolating *V. kanaloae* strain 1 C and purifying marine EVs was confirmed through metagenomic fragment recruitment (Supporting Information S1: Figure. S5). *V. coralliilyticus* is a common coral pathogen (Rubio‐Portillo et al. 2020, 2014), while different strains of *V. kanaloae* have been linked to disease in oysters and clams (Huang et al. 2021). One of our objectives was to investigate the potential interaction and uptake of fluorescently labeled EVs derived from the aforementioned *Vibrio* spp. by cells from other bacterial recipient taxa using the fluorophores AF‐488 and FM1‐43 (Supporting Information S1: Table S1). This aimed to determine whether taxonomy (i.e., phylogenetic distance) serves as a boundary for EVs uptake. We excluded the fluorophore FM4‐64 because its weak membrane‐binding and loose association with EV membranes, which made it unsuitable for our study (Supporting Information S1: Figure S6). In contrast, the combination of fluorophores AF‐488 and FM1‐43 demonstrated that, after three Amicon washes, the dyes remained associated with the vesicles, exhibiting higher fluorescence compared to the stained and washed HEPES buffer (Supporting Information S1: Figure S4, panel D, *p*‐value = 2.561e‐06). It is important to note that after fluorescently labeling, EVs were thoroughly washed before being added to bacterial cultures to remove any residual free dye from the EVs solution, which could otherwise bias the results.

**Figure 1:**
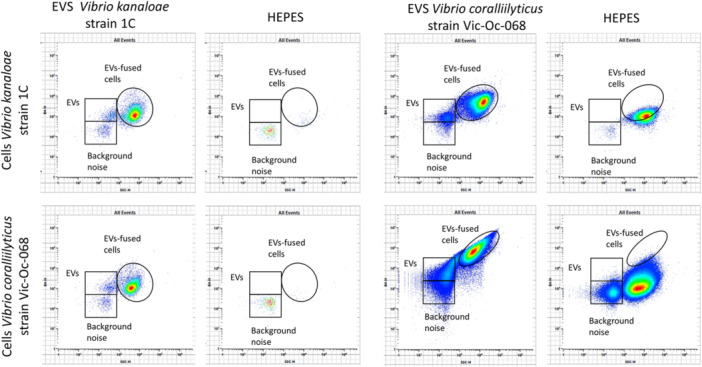
Flow cytometry analysis of fusion experiments. The plots show the fusion of EVs labeled with Alexa Fluor 488 and FM 1‐43 from *Vibrio kanaloae* with its own cells, as well as with *Vibrio coralliilyticus* strain Vic‐Oc‐068 cells (left panels), and the reciprocal experiment (third column). Each fusion experiment plot (EVs) includes its corresponding negative control (HEPES). Negative controls were conducted using the same cell and HEPES buffer (instead of EVs; dyed and washed) volumes as the positive fusion experiments, ensuring comparable conditions for all assays. Both types of EVs appear to fuse with the bacterial cells, leading to a shift in the bacterial population.

The uptake and potential fusion of fluorescently labeled EVs with recipient cells was monitored using spectral flow cytometry, which captures the full fluorescence spectrum for the marker dyes across all laser lines used in our experiments. Previously, conventional flow cytometry with lower resolution for detecting nanoparticles showing low fluorescence signal has been used to monitor fusion of marine EVs to cells (Biller et al. 2022). EVs fusion to cells was inferred when the bacterial cell population incubated with stained EVs increased their fluorescence signal and thus shifting the position in the cytometer plot, while the side scatter values for the recipient cell population remained very similar (Biller et al. 2022). The use of different negative controls (see methods) aided at the discrimination of positive labeled recipient cells (Supporting Information S1: Figure S7). After incubation of labeled EVs with different bacterial cells (seven different bacterial species; see Supporting Information S1: Table S1), the spectral signature of dyes showed a higher intensity in the recipient bacterial cell population compared with controls, indicating that labeled EVs have been likely fused with cell membranes incorporating part of the fluorescence EVs signal into the cell (Figure 1; see green fluorescent channel labeled as “BH4”). The resolution of spectral flow cytometry was high enough to distinguish labeled free (non‐fused) EVs based on size scatter and fluorescence signal (Figure 1 and Supporting Information S1: Figures S4 and S8).

**Figure 2:**
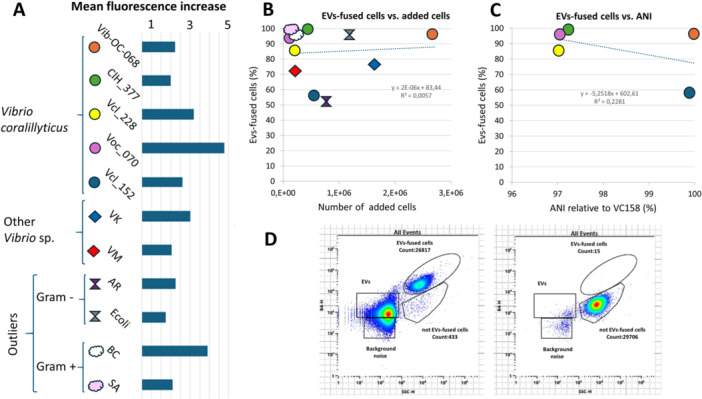
Results of the fusion experiments of different cells with *Vibrio coralliilyticus* strain Vic‐Oc‐068 EVs. For the fusion experiments, five different strains of *Vibrio coralliilyticus* (VC) were used (Vic‐Oc‐068, ClH_377, Vcl_228, Voc_070 and Vcl_152; circle), other species of *Vibrio* (diamond shape) such as *V. kanaloae* (VK) and *V. mediterranei* Vib‐OC‐097 (VM), Gram negative outliers (ribbon shape) such as *Arcobacter roscoffensis* (AR) *and Esherichia coli* (Ecoli) and Gram positive outliers (cloud shape) such as *Bacillus cereus* (BC) and *Staphylococcus aureus* (SA) were incubated with EVs *of Vibrio coralliilyticus* Vic‐Oc‐068 and analyzed using cytometry. The increase of mean fluorescence in the gate of EVs‐fused cells was measured dividing the mean fluorescence in the sample (EVs dyed and washed + cells) by the mean fluorescence of the negative control (HEPES dyed and washed + cells) (A). The percentage of EVs‐fused cells was compared with the number of added cells (B) and the ANI of the strains of *Vibrio coralliilyticus* used in this experiment relative to the *Vibrio coralliilyticus* strain Vic‐Oc‐068 (C). Example of the *Vibrio coralliilyticus* ClH_377 fusion with EVs (D). The data was obtained comparing the mean fluorescence and count in the EVs‐fused cell gate in the sample (EVs dyed and washed + cells, D left) and the negative control (HEPES dyed and washed + cells, D right). These percentages should be interpreted with caution, as there is a possibility that we overestimated the number of cells due to potential cell division triggered by the addition of EVs.

Flow cytometry data revealed EV fusion events not only between bacteria of the same species but also across different taxa. As observed in the plots of *V. coralliilyticus* strain Vic‐Oc‐068 versus *V. kanaloae* strain 1 C since both *Vibrio* species were able to fuse and uptake their own vesicles and the vesicles from the other *Vibrio* species (Figure 1). Data showed that a high percentage of the *V. coralliilyticus* strain Vic‐Oc‐068 population (97.3%–99.41%) fused with both types of *Vibrio* EVs tested, whereas 77% of *V. kanaloae* cells fused with EVs from *V. coralliilyticus* strain Vic‐Oc‐068. In all cases, the mean fluorescence intensity within the EVs‐fused cell gate was greater in the sample compared to the control (Figure 2A; 1.71‐2.56‐fold change; Supporting Information S1: Table S2). Fusion was also corroborated using confocal microscopy (Supporting Information S1: Figure S9).

We next explored the fusion potential of *V. coralliilyticus* strain Vic‐Oc‐068 EVs—which exhibited greater variability in the percentage of fused cells than *V. kanaloae* EVs—with a broader range of bacteria, including other *V. coralliilyticus* strains, *Vibrio mediterranei*, and *Arcobacter roscoffensis*, as well as non‐marine Gram‐negative and Gram‐positive species such as *Escherichia coli*, *Bacillus cereus*, and *Staphylococcus aureus*. The availability of *V. coralliilyticus* strains from distinct Mediterranean locations including Genova (Voc_070) and Alicante (other *V. coralliilyticus* strains used in this paper) allowed us to assess whether geographic origin or genomic similarity (measured by Average Nucleotide Identity, ANI) correlated with EV–cell fusion efficiency. According to our data all tested bacteria were able to potentially uptake EVs from *V. coralliilyticus* strain Vic‐Oc‐068 (Figure S9). A marked increase in mean fluorescence intensity was observed in the EVs‐fused cell gate for the samples compared to the control, with an increased fluorescence signal from 1.23‐ to 4.27‐fold (Figure 2A). Data also indicated that the percentage of bacterial cells that potentially uptake EVs was between 53% and 99%. For instance, more than 90% of cells in the bacterial culture showed fluorescence compatible with *V. coralliilyticus* Vic‐Oc‐068 EVs fusion in 6 out of the 11 different bacterial taxa tested (Figure 2B). No correlation was found between added cell numbers and the EVs uptake across all taxa (*R* ^2^ < 0.1; Figure 2B), not even when the comparison was made between fusion experiments that used the same amount of EVs against different bacteria (Supporting Information S1: Figure S10; in which the cytometer plots shown are representative from technical replicates, Supporting Information S1: Figure S11). *V. coralliilyticus* strain 377 showed an EVs‐fused cell percentage of 99.44% with a ratio of 587 EVs/cell, whereas *V. mediterranei* displayed a lower percentage of EVs‐fused cells (73.05%) despite a higher EVs‐to‐cell ratio of 1120. Serial dilutions of stained and HEPES‐washed EVs facilitated accurate EVs quantification via flow cytometry in this experiment (Supporting Information S1: Figure S8).

The percentage of *V. coralliilyticus* Vic‐Oc‐068 EVs fused with recipient cells did not correlate with taxonomy or phylogenetic distance. Outliers such as *B. cereus, S. aureus*, and *E. coli*—taxonomically distant from *Vibrio* spp.—had high percentages of EVs‐fused cells (> 97%), while some *Vibrio* spp. recipients exhibited lower fusion rates despite being from the same genus (Figure 2B). To examine whether intraspecific genetic similarity influenced EVs uptake, we compared the ANI with the percentage of EVs‐fused cells in five *V. coralliilyticus* strains and found no correlation (*R*² = 0.23; Figure 2C). Spatial distribution also appeared irrelevant, as fusion rates were higher between two geographically distant strains (Vic‐Oc‐068 and Voc_070, both isolated from *Oculina patagonica*) than between strains collected from the same site (e.g., Vic‐Oc‐068 and Vcl‐228; Supporting Information S1: Figure S10).

Tashiro et al (Tashiro et al. 2017). found that EVs from *Buttiauxella agrestis* interacted more specifically with the parent strain cells compared to other tested strains. Biller et al (Biller et al. 2022). incubated EVs from *Prochlorococcus* with different marine cells in mid‐exponential growth phase, proving that those EVs associate with different marine cells, including Pelagibacter (Biller et al. 2022). In our study, we also observed differences in the interaction and fusion of the same EVs with different recipient species. While some degree of stochasticity cannot be excluded, the different percentage of EVs‐fused cells using the same number of vesicles (Supporting Information S1: Figure S12) suggest that these interactions were unlikely to be purely random. Instead, other factors may help explain these differences, including characteristics of the cell's envelope structure, surface charge (Tashiro et al. 2017), membrane hydrophobicity (MacDonald and Beveridge 2002) or nutrient status of the recipient cell. Indeed, it has been described that the EVs‐microbial interaction depends on the nutrient conditions of the culture (Warsi et al. 2023). In our case, the cultured cells were collected at the stationary phase (see methods) and resuspended in HEPES buffer lacking nutrients. Consequently, the cells might have used the EVs as a nutrient resource, potentially serving as carbon providers, as observed by Biller et al (Biller et al. 2014; Biller et al. 2022; Biller et al. 2017). To test this hypothesis, 100 μL of cells from *V. coralliilyticus* strain Vic‐Oc‐068 suspended in HEPES buffer (initial OD_600_ = 0.39 ± 0.006; three technical replicates) were mixed with 200 μL of fluorescently labeled and washed *Vibrio* EVs and incubated for 1 h at 30°C. Optical density measurements taken after 1 h of incubation with EVs showed increased cell growth, (OD600 = 0.766 ± 0.01; three technical replicates) consistent with previous observations in the literature. The percentages of cells that fused with EVs discussed above should be interpreted with caution, as these values may be overestimated if EVs were used as a carbon source and allowed cells to undergo one round of division during the 1‐h incubation.

Some of the EV‐cell interactions observed in our study may reflect natural ecological relationships. For instance, the interaction between *Vibrio mediterranei* Vib‐OC‐097 and *V. coralliilyticus* strain Vic‐OC‐068 ‐both originally isolated from the same *Oculina patagonica* coral specimen (Rubio‐Portillo et al. 2014)—appears to enhance their pathogenicity toward *O. patagonica* when they interact (Rubio‐Portillo et al. 2020). Given that one of the primary functions attributed to EVs is intercellular communication (Mashburn and Whiteley 2005), the observed interaction between EVs from *V. coralliilyticus* strain Vic‐OC‐068 and *V. mediterranei* cells suggests a potential mechanistic basis for previously documented ecological interactions, such as coral infection (Rubio‐Portillo et al. 2020).

### 3.3 EVs Dynamics, DNA Cargo and Sequencing of Vesicular DNA in *Vibrio* spp.

**Figure 3:**
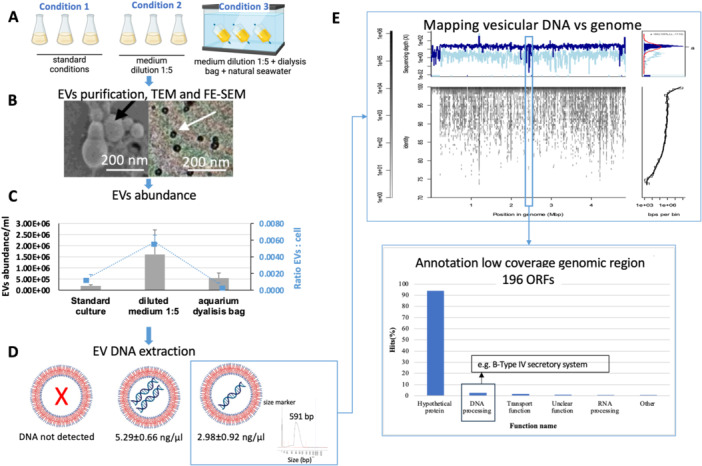
EV experiment from *Vibrio kanaloae*. (A). Experimental design. *V. kanaloae* strain 1 C was growth in three different conditions: standard one, medium diluted 1:5, and in dialysis bag containing medium diluted 1:5 placed in aquarium with natural seawater from the same sampling point in which the strain was isolated. (B). Image of negative staining and FE‐SEM of EVs purified by Optiprep gradient from *V. kanaloae* strain 1 C. (C) EVs concentration (top) and EVs‐to‐bacterial cell ratios (bottom) in *V. kanaloae* strain 1 C cultures under the different incubation conditions. Standard deviation is provided. EVs abundance was performed using a NanoSight NS300 instrument (Malvern Panalytical, Malvern, UK). EV:cell ratio was calculated by normalizing EVs concentrations to bacterial concentrations for each sample. Bacterial cells were quantified using DAPI staining. (D) DNA concentration and size distribution obtained from EV fraction from the different conditions. Extracted DNA was likely under the detection limit from standard conditions or either DNA was not exported at all under this condition. Experiment was repeated twice with identical results. Fragment size distribution was performed using the 5300‐Fragment analyzer, and a kit tailored for High Sensitivity (HS) Large Fragment analysis with a target size of 50 kb. The two peaks observed at 1 bp and 10,000 bp are internal size standards. Unfortunately, we encountered challenges in measuring the size distribution of DNA from the diluted culture conditions (Figs, 3 A and 3D) using a Bioanalyzer instrument despite the measurement experiments were repeated at the technical service of the University of Alicante. (E) Mapping of quality filtered Illumina reads obtained from the 20% EVs DNA fraction of aquarium dialysis bag cultures to *V. kanaloae* strain 1 C genome. Two replicates were sequenced with nearly identical mapping results and genome coverage pattern. The results depict the alignment of quality‐filtered Illumina raw reads from replicate 1, the same was obtained from second replicate. Mapping was conducted using Enveomics bioinformatic package. Gene annotation for the region showing low genome coverage is shown in bottom panel. The annotation was performed using geNomad program (Camargo et al. 2023).

In the EVs fusion experiments discussed above, the highest rates of EV‐fused recipient cells (97%–99%) were observed for *V. kanaloae* strain 1 C, indicating efficient vesicle uptake by various bacterial species. Since nutrient conditions, as discussed above, is likely an important factor contributing on EVs production and type of packaged cargo (e.g. DNA molecules), we therefore used *V. kanaloae* strain 1 C as a model in a microcosm experiment with different culture conditions (Figure 3A) as follows: (1) standard culture conditions (see methods), (2) nutrient dilution conditions (DifcoTM Marine Broth 2216 medium was diluted 1/5 to simulate more oligotrophic conditions similar to that of the Mediterranean Sea), and (3) nutrient dilution as condition No. 2 but incubating the culture inside dialysis bags in an aquarium filled with natural seawater collected from the same sampling point in which *V. kanaloae* strain 1 C was originally isolated (Figures 3A and 3B). Released EV concentrations (1 × 10^5^ – 1.6 × 10^6^; ratio of < 6 EVs per 1000 cells; Figure 3C) did not significantly differ across nutrient conditions (ANOVA; *p*‐value of 0.213).

Over half of the DNA in the open ocean is present outside living organisms (Linney et al. 2022), such as extracellular DNA or in EVs. This extracellular DNA represents a substantial source of nutrients for microbes; not to mention that it is also a large amount of DNA potentially available for recombination and horizontal gene transfer (Lücking et al. 2023). Thus, we decided to investigate whether DNA cargo varied across nutrient conditions. No measurable DNA was detected under standard culture (Condition 1, triplicates), implying vesicle DNA levels were below detection limits. In contrast, in the other two conditions, No. 2 and No. 3, which correspond to the nutrient diluted conditions (Figure 3D and Table S3), vesicular bulk DNA concentrations varied from 2.98 to 5.29 ng/μL, respectively. The difference between these two conditions was statistically significant (ANOVA; *p*‐value = 0.02). The mean DNA fragment length from aquarium‐grown cultures (Condition 3) was 591 bp, spanning 250–1440 bp (Figure 3D). Studies have shown that genomic content in EVs can reach up to 370 Kbp in size, with smaller DNA fragments being more frequently reported within microvesicles (MVs) (Domingues and Nielsen 2017; Klieve et al. 2005; Chiura et al. 2011; Dorward et al. 1989). DNA in EVs has been extensively documented, with evidence suggesting chromosomal, plasmid, and phage origins (Domingues and Nielsen 2017; Orench‐Rivera and Kuehn 2016). We speculate that under nutrient‐diluted conditions, *V. kanaloae* strain 1 C may experience increased physiological stress. Such stress conditions have been associated with enhanced extracellular vesicle production in bacteria (Mozaheb and Mingeot‐Leclercq 2020), along with the incorporation of intracellular components, including DNA.

We then explored the genomic content of EVs released by *V. coralliilyticus* strain Vic‐Oc‐068 (used in EV fusion assays) and *V. kanaloae* strain 1 C (aquarium Condition 3; Tables S4, S5), to determine whether certain genomic elements are preferentially packaged. In the case of vesicular DNA from *V. kanaloae* strain 1 C, sequencing data indicated (Figure 3E) that nearly all genome of *V. kanaloae* (4.8 Mbp; sequenced by HiFi PacBio; Supporting Information S1: Tables S6 and S7) was exported in vesicles, except for a 129 kb underrepresented region (*n* = 196 ORFs) was underrepresented in the EV fraction (Figure 3E; top panel). This region likely corresponded to a plasmid‐phage element according to gene annotation (B‐Type IV secretory system along with phage integrase‐like and high proportion of hypothetical proteins; Supporting Information S1: Table S8), as described by Rocha & Bikard (Rocha and Bikard 2022).

Regarding *V. coralliilyticus* strain Vic‐Oc‐068, the mapping of sequenced reads obtained from vesicular DNA showed that reads were randomly recruited against the parent genome (Supporting Information S1: Figure S13 and Table S9). A significant proportion of reads did not match *V. coralliilyticus* (Supporting Information S1: Table S10), likely due to contamination. Additionally, multiple displacement amplification (MDA) may have introduced biases, affecting the representation of genomic regions in the amplified DNA. As a result, we could not confirm specific genome regions preferentially packaged into EVs. Similar to *V. kanaloae* strain 1C, other studies ‐e.g. *Thermococcus onnurineus* NA1T (Choi et al. 2015) and marine *Prochlorococcus* (Biller et al. 2017; Choi et al. 2015)‐ have shown vesicle DNA representing widespread genomic segments, covering over 50% of the source genome.

## 4 Conclusions and Study Limitations

This study optimized the fluorescence labeling of marine EVs from different species of *Vibrio* spp. using the fluorophores AF‐488 and FM1‐43, enabling their detection through confocal and super‐resolution microscopy, as well as spectral flow cytometry, with negative controls showing negligible signals. Findings revealed that EVs from marine *V. coralliilyticus* can fuse with a broad range of bacterial taxa, including both Gram‐negative and Gram‐positive species, irrespective of phylogenetic distance. This suggests that EV uptake is not governed by phylogenetic distance between the EV donor and recipient, and may instead be influenced by other factors, such as nutrient status. We also investigated how nutrient conditions affect the DNA content within EVs, highlighting that under nutrient‐diluted conditions, *V. kanaloae* strain 1 C released vesicles containing measurable DNA, whereas standard nutrient conditions yielded undetectable DNA levels in EVs. Sequencing revealed that the vesicular DNA encompassed nearly the entire genome, except for an underrepresented genomic region of 129 kb, likely associated with a plasmid‐phage element. Collectively, this study highlights the versatility of EV‐mediated interactions among marine bacteria and underscores the variability of DNA cargo in EVs under different nutrient scenarios within marine ecosystems.

## Author Contributions

**Nadefa Adda Nekrouf:** investigation, writing – original draft, methodology, visualization, writing – review and editing, formal analysis, software, data curation. **Lucia Maestre‐Carballa:** investigation, methodology, writing – original draft, writing – review and editing, visualization, software, formal analysis, data curation. **Monica Lluesma‐Gomez:** investigation, methodology, writing – review and editing. **Esther Rubio‐Portillo:** investigation, writing – review and editing, methodology, validation. **Manuel Martinez‐Garcia:** conceptualization, investigation, funding acquisition, writing – original draft, writing – review and editing, visualization, validation, methodology, software, formal analysis, project administration, resources, supervision, data curation.

## Ethics Statement

The authors have nothing to report.

## Conflicts of Interest

The authors declare no conflicts of interest.

## Supporting information

Supporting File

## Data Availability

The data that support the findings of this study are openly available in Genbank at https://www.ncbi.nlm.nih.gov/bioproject/, reference number PRJNA1217409. The datasets generated and analyzed during the current study are available in the Genbank Bioproject number PRJNA1217409.
