# Supplementary material for: Unveiling Marine Vesicle Uptake in Vibrio spp: Taxonomic and Environmental Insights
Source: Microbiologyopen. 2026 May 10;15(3):e70311. doi: 10.1002/mbo3.70311 (PMC13158281; doi:10.1002/mbo3.70311)
Supplement: Supplementary file 1 — Supporting File [file MBO3-15-e70311-s001.docx]

**Supplementary Material**

**Unveiling marine vesicle uptake in *Vibrio* spp: Taxonomic and Environmental Insights**

Nadefa Adda Nekrouf^1,2,3^, Lucia Maestre-Carballa^1,2^, Monica Lluesma-Gomez^1,2^, Esther Rubio-Portillo^1^, and Manuel Martinez-Garcia^*1,2^

^1^Department of Physiology, Genetics, and Microbiology, University of Alicante, Carretera San Vicente del Raspeig, San Vicente del Raspeig, Alicante, 03690, Spain

^2^Multidisciplinary Institute for Environmental Studies (IMEM), University of Alicante, Carretera San Vicente del Raspeig, San Vicente del Raspeig, Alicante, 03690, Spain

^3^University Mustapha Stambouli, Mascara, 29000, Algeria

Underlined authors have contributed equally to this manuscript

**This file contains 13 supplementary figures, 10 supplementary tables, and references**


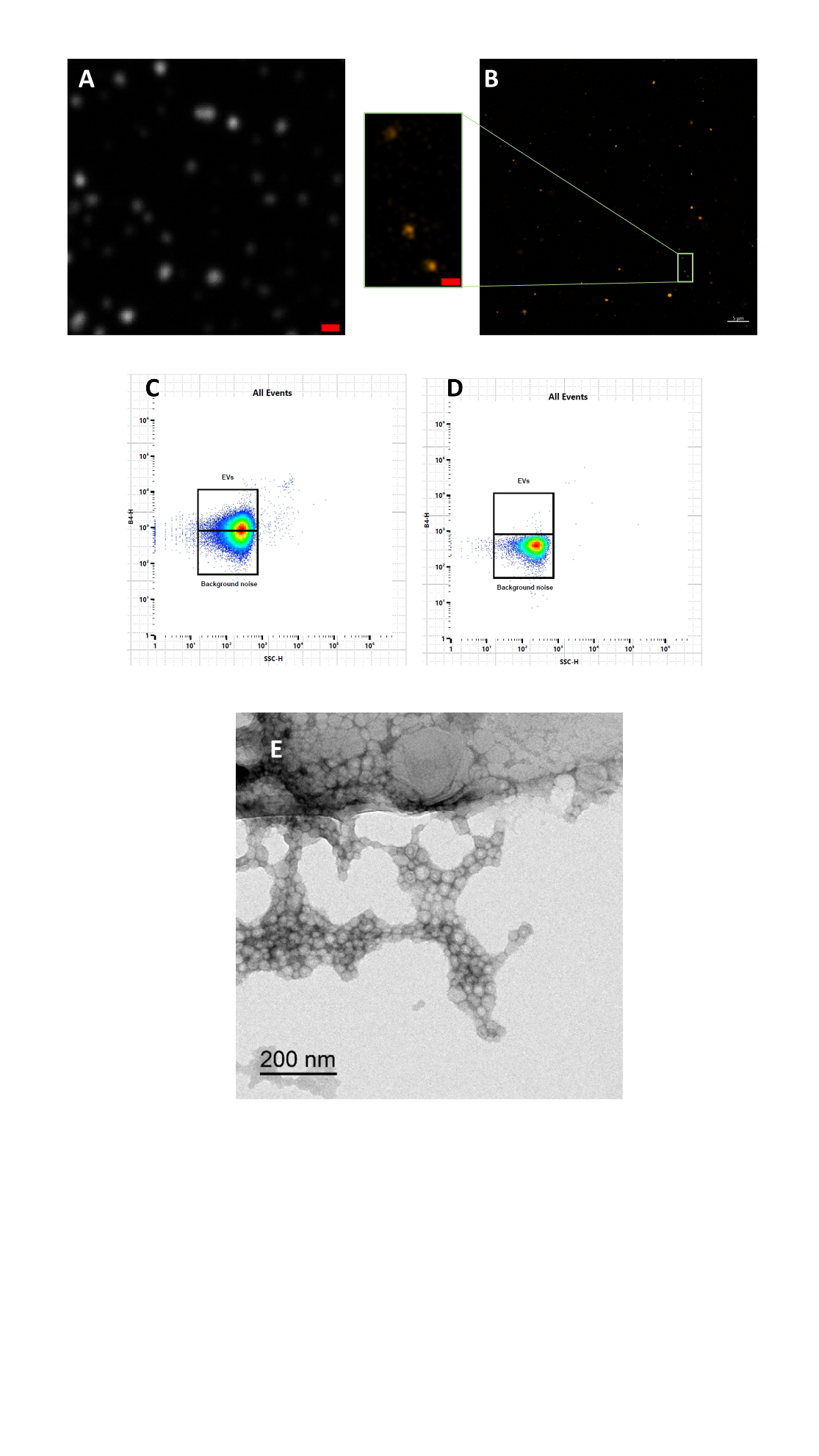


**Fig. S1.** EVS dye protocol was successfully used for the detection of vesicles of *Vibrio coralliilyticus* strain Vic-Oc-068 with different methodologies: for (A) super-resolution microscopy (c:2/2, z:19/36, t:1/4), (B) confocal microscopy and (C and D) flow cytometry. A portion of panel B has been scaled to better facilitate comparison among microscopy images. The cytometry plot on the left shows EVS stained of *Vibrio coralliilyticus* strain Vic-Oc-068 (EVs gate) (C), while the plot at the right show the same sample filtered through 0.02 μm, which removed the vesicles (D). Red bar represents 570 nm. (E) Vesicles of *Vibrio coralliilyticus* strain Vic-Oc-068 were observed using TEM.


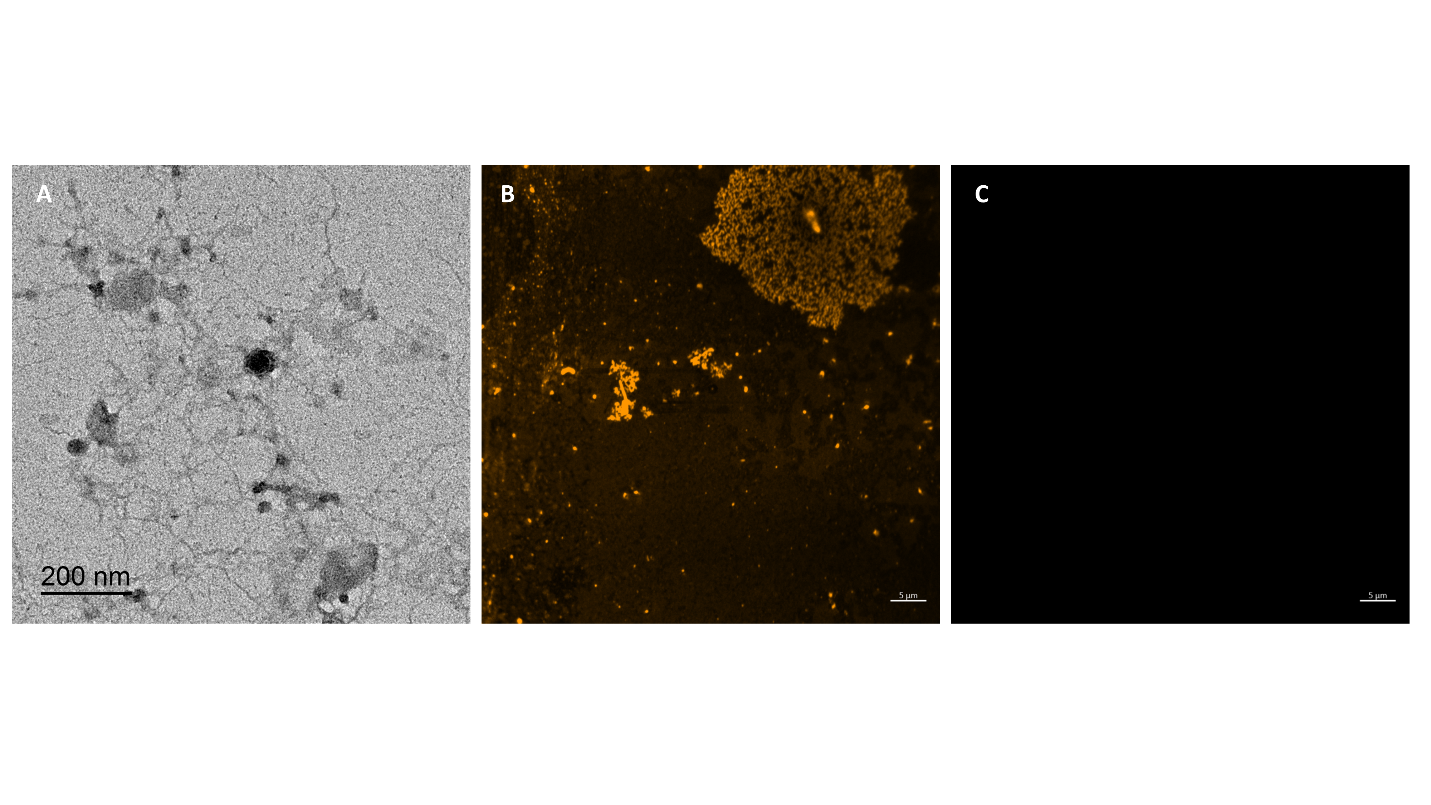


**Fig. S2.** Vesicles images obtained from sea water. EVS from 25 L of sea water (filtered through 0.2 μm) were concentrated with Vivaflow cartridge and purified using Optiprep gradient. (A) TEM was used to confirm the presence of vesicles and absence of virus in the optiprep fractions 20 and 25%. (B) Sea water vesicles were observed after labeling them for an hour with Alexa Fluor 488 and FM 1-43, followed by 3 washes with 5 mL of HEPES each. (C) The negative control consisted in HEPES treated as vesicles were treated.


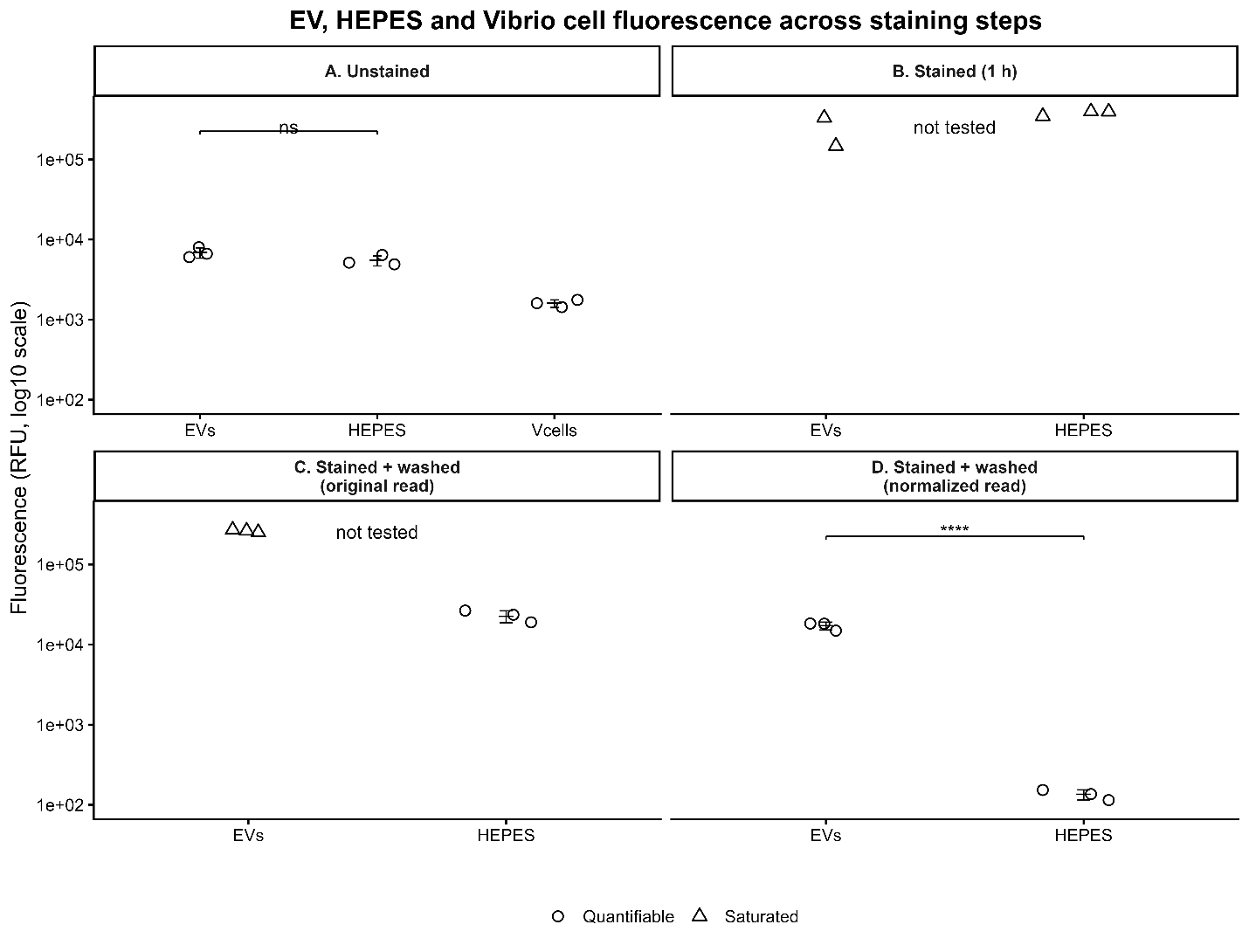
**Fig. S3.** Fluorescence was measured using CLARIOstar (BMG LABTECH, Germany) in unstained samples of HEPES, EVs and cells of *Vibrio coralliilyticus* strain Vic-Oc-068 (A). EVs ad HEPES were then stained with Alexa Fluor 488 and SynaptoGreen C4 for 1 hour and measured after the incubation time (B). After three washes with Amicon, EVs and HEPES fluorescence was measured using the original acquisition settings (C), and normalized acquisition settings (D). Each point represents one biological replicate (mean of three technical measurements). Open circles indicate quantifiable values and open triangles indicate saturated values (≥260,000 RFU). Horizontal bars show means and error bars indicate standard deviation. Statistical comparisons between EVs and HEPES were performed only for quantifiable data using Welch’s t-test on log10-transformed RFU values.

**
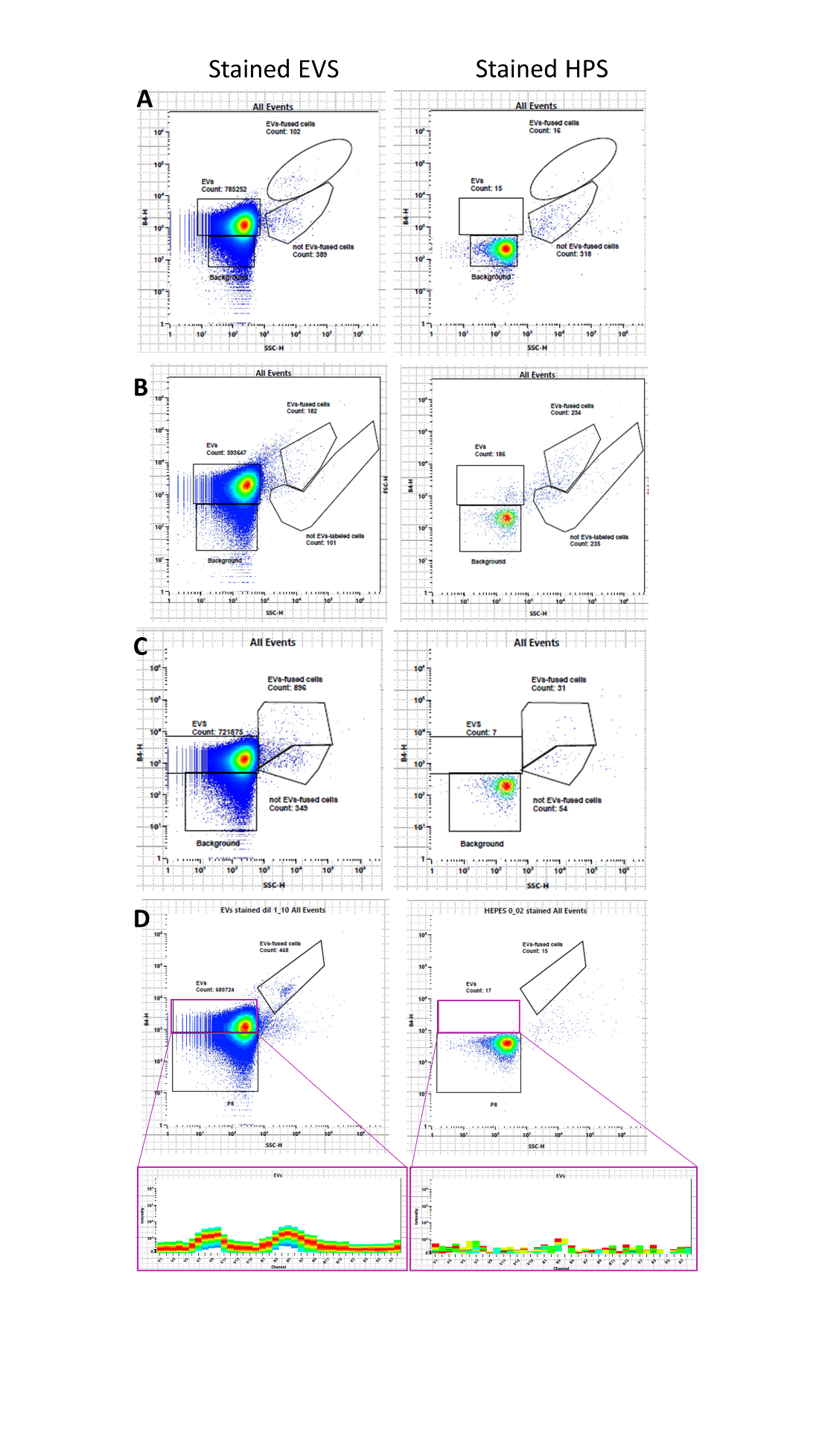
**

**Fig. S4**. Cytometer plots of stained EVs and HEPES. EVs of *Vibrio coralliilyticus* strain Vic-Oc-068, and HEPES were both dyed with Alexa Fluor 488 and FM 1-43 for one hour, followed by 3 washes with 5 mL of HEPES, and then analyzed using a flow cytometer. Unless otherwise indicated, the concentration of EVs was 1/1, matching that of the HEPES control. Each panel (A-D) represents an independent experiment. Aurora Spectral Cytometer (Cytek) allowed us to explore the intensity within the EVs gate, revealing distinct patterns for HEPES and EVS dyed samples (D).

**
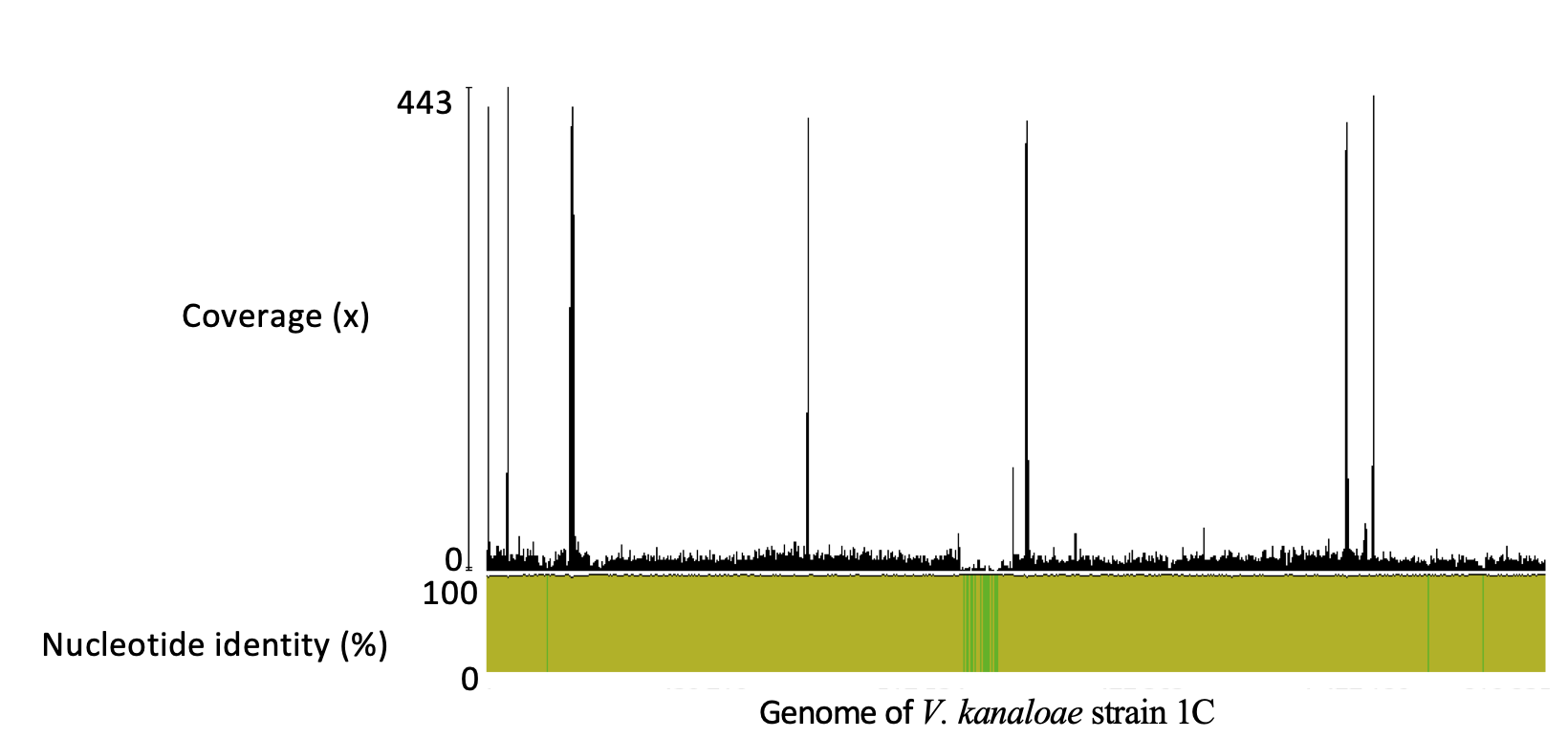
**

**Fig. S5.** Mapping of *V. kanaloae* strain 1C genome against microbial metagenomes [1] obtained from natural seawater collected from the same sampling point in which this species was originally isolated. Mapping was performed with Bowtie 2.0 in Geneious bioinformatic package using default parameters. Genome of *V. kanaloae* strain 1C is represented in X axis. After mapping, black histograms represent sequencing coverage or read mapping from 0-443X. Y axis represent coverage (X). Nucleotide identity of read mapping is shown. Most of the reads showed nearly 100% of nucleotide identity with genome of *V. kanaloae* strain 1C. Light green color depicts region with no mapping or very low mapping coverage.

**
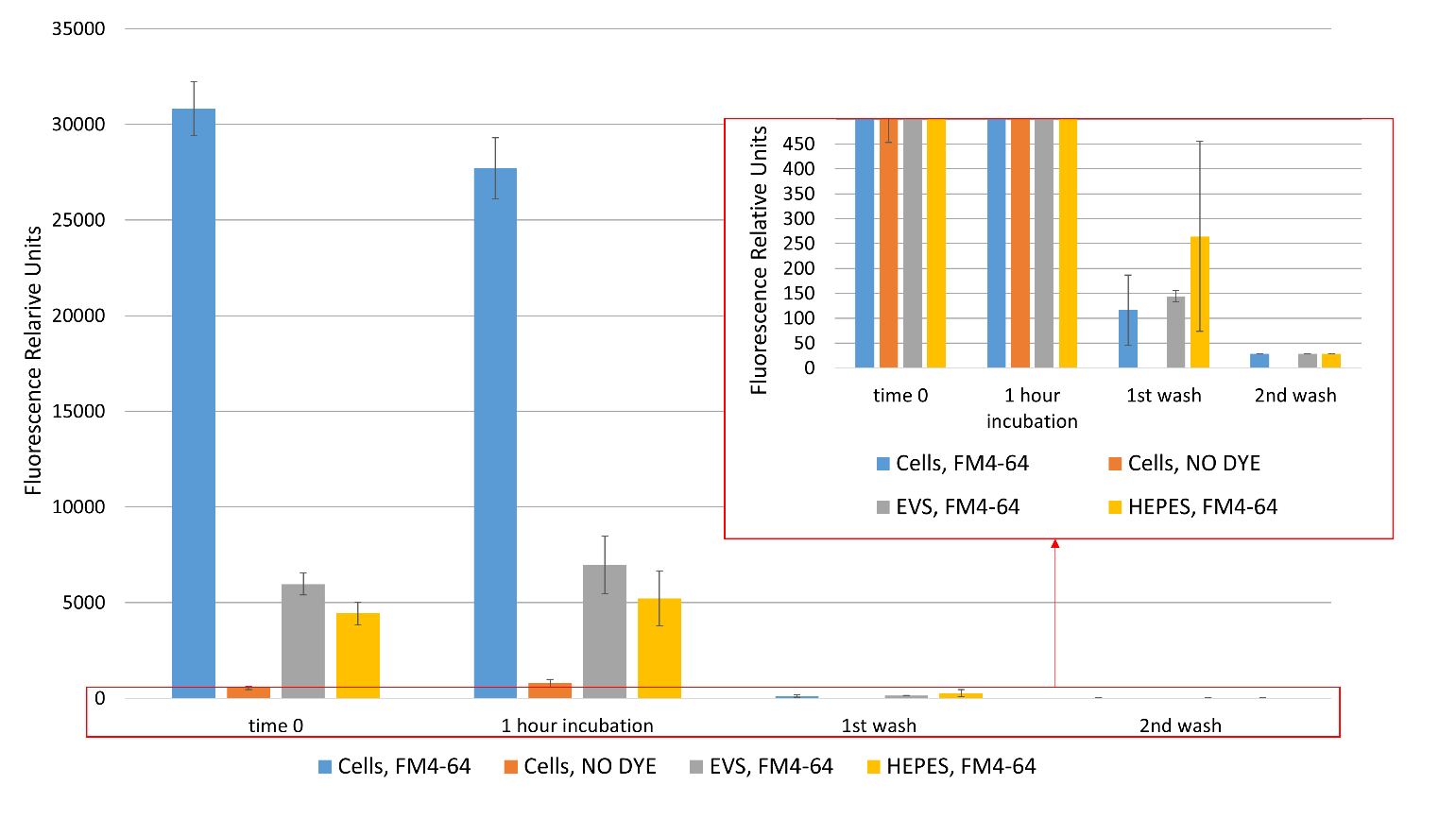
**

**Fig. S6.** Fluorescence labelling with FM4-64 and the effect of 2 washes. Cells (blue) and EVs (grey) from *Vibrio kanaloae* 1C were stained with FM4-64 and subsequently washed two times using an Amicon membrane and HEPES buffer. The fluorescence was measured with CLARIOstar (BMG LABTECH, Germany) plate reader as soon as the fluorophore was added (time 0), after 1 hour of incubation, and after the first and the second wash. A negative control, consisting of untreated cells was also included (Cells, NO DYE; orange). In addition, HEPES buffer (yellow) was also dyed with FM4-64, and processed as the other samples. Each measurement was performed in duplicate. SD is also represented.


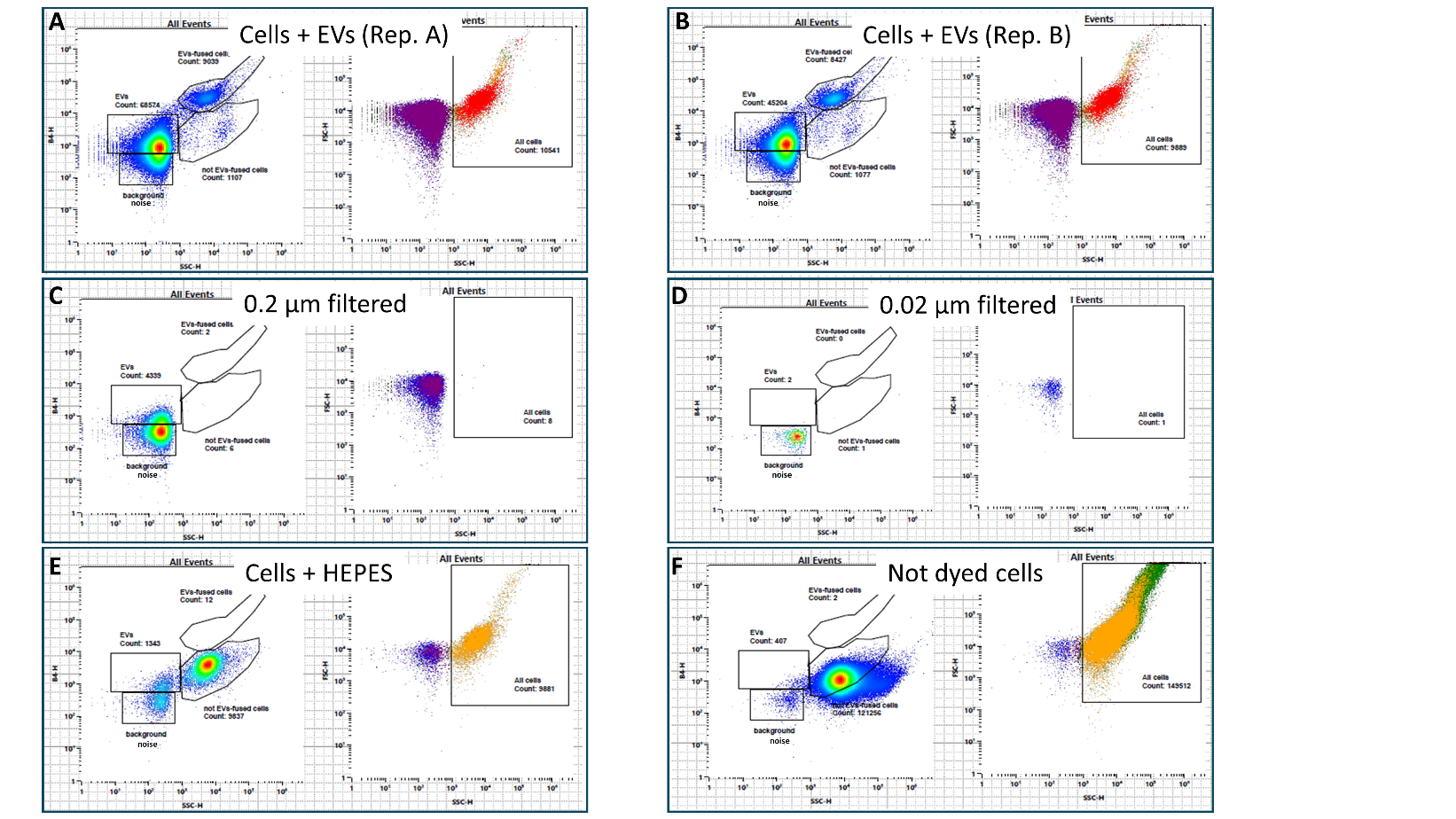


**Fig. S7.** Cytometry plot of *Vibrio coralliilyticus* strain Vic-Oc-068 EVS vs. *Vibrio coralliilyticus* Vcl_228 cells. Both fluorescence at BH4 channel (left) and FFSC-H (right) were represented against SSC-H. Cell gates (EVs-fused cells and no EVs-fused cells at the left and all cells at the right) of replicates (A and B) were defined after filtering the sample with a 0.2 µm filter, which effectively removed the cells, showing just EVs in the EVs gate (C). The gate EVs corresponded to vesicles, which were removed upon filtering with a 0.02 µm filter (D). EVs-fused cells gate was established by comparing the sample (Cells+EVs) with the negative control (Cells+HEPES) (E) that represented the non EVs-fused cells or the effect of the fluorophores on their own. For comparison we also analysed the cells without labelling (F). All cells gate from FFSC-H vs. SSC-H plot was used to count the total amount of cells added to the experiment, while the EVs-fused cells gate was used to count the amount of cells that fused with EVs.


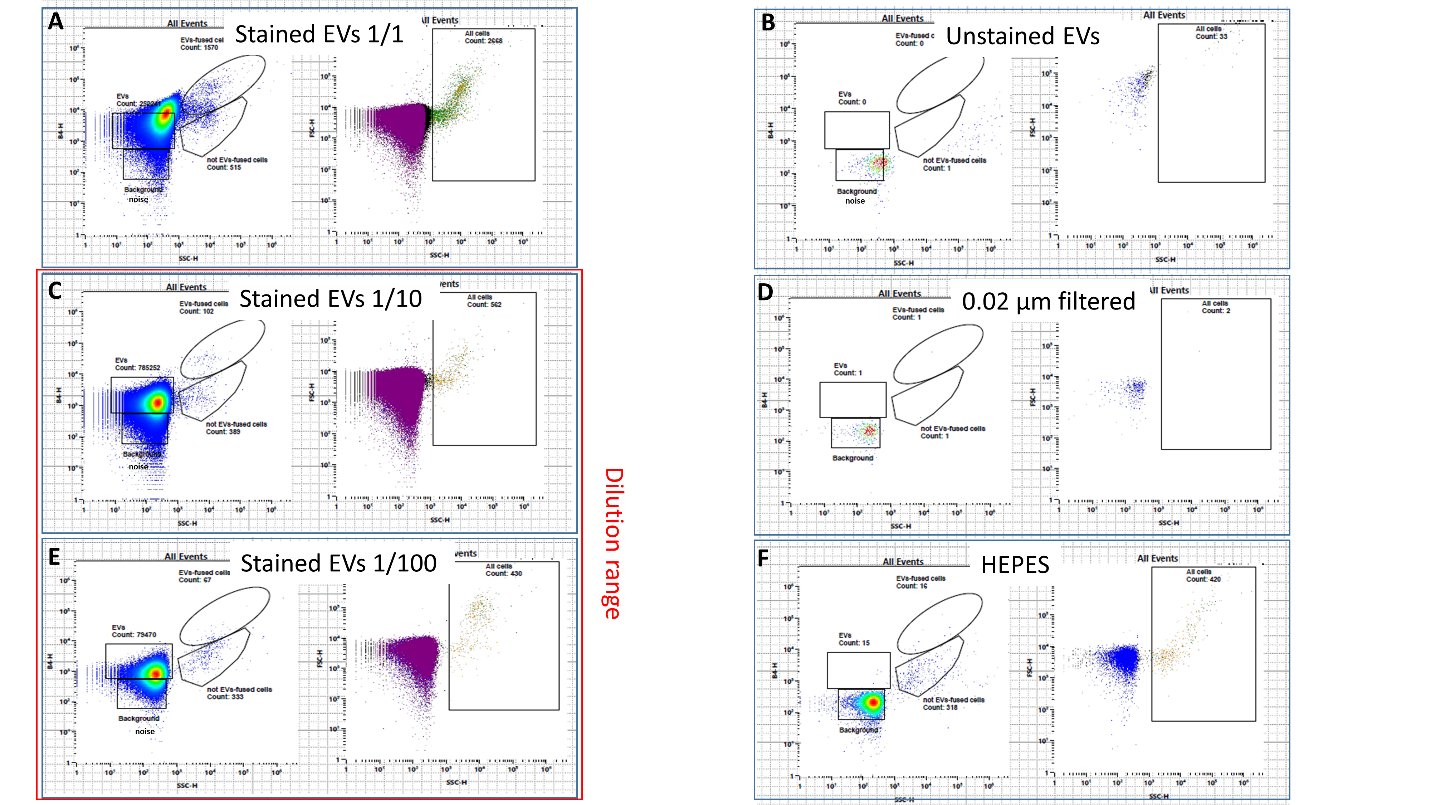


**Fig. S8**. Cytometry plot of *Vibrio coralliilyticus* strain Vic-Oc-068 EVS dyed with AF-488 and FM1-43. Both fluorescence at BH4 channel (left) and FFSC-H (right) were represented against SSC-H. Even though the SSC-H vs. FFSC-H representation did not facilitate EVs quantification, green fluorescence (BH4) vs. FFSC-H enabled EVs accurate numeration. To quantify the vesicles, serial dilutions of the EV sample (A) were conducted until the observed vesicle counts corresponded proportionally to the dilution factor (C and E), thereby minimizing swarm detection and ensuring reliable measurements. EVs gate was defined using the 0.22 μm filter (removes bacteria) and 0.02 μm filter (removes vesicles; D). HEPES dyed and washed (F) as the EVs sample was analyzed with cytometry to subtract non EV-artifacts within the EV gate.

**
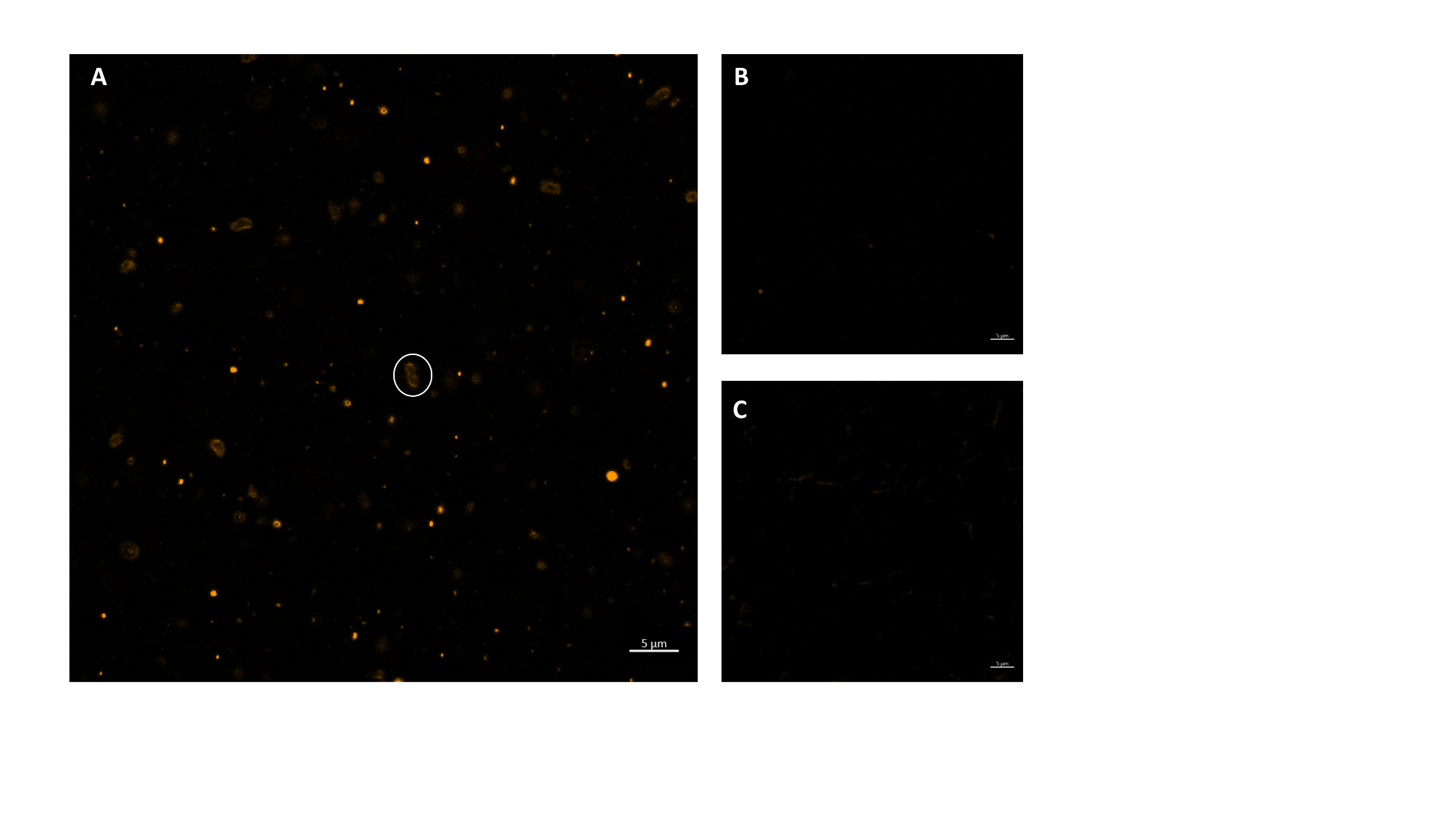
**

**Fig. S9.** Confocal images of EVs-bacteria fusion. (A) *Vibrio coralliilyticus* strain Vic-Oc-068 EVs labeled with FM1-43 and Alexa Fluor 488 after 1-hour incubation with *Vibrio coralliilyticus* cells (white circle). (B) *Vibrio coralliilyticus* EVs labeled with FM1-43 and Alexa Fluor 488 filtered through 0.02 um membrane filter, and later incubated for 1-hour with *Vibrio coralliilyticus* cells. (C) Negative control: HEPES (10 mM, 0.85% NaCl) dyed with FM1-43 and AF 488 and washed with an Amicon filter. Scale bar: 5 μm. In this experiment, a culture of *Vibrio coralliilyticus* strain Vic-Oc-068 was incubated with purified EVs previously labelled with FM1-43 and AlexaFluor 488*.* After incubation time, the cells showed up an apparent fluorescence signal in which the cell morphology and shape could be clearly distinguished. In addition, no cell fluorescence signal was observed in controls depicted in panels B and C in which 1) cells were incubated following the same procedure as sample but with HEPES buffer containing the same concentration of fluorochromes (C) and 2) stained EVs previously filtered by 0.02 micrometers prior to incubation (B). According to data, it seems that fluorescence of EVs has been transferred to the membrane of receipt cells.

**
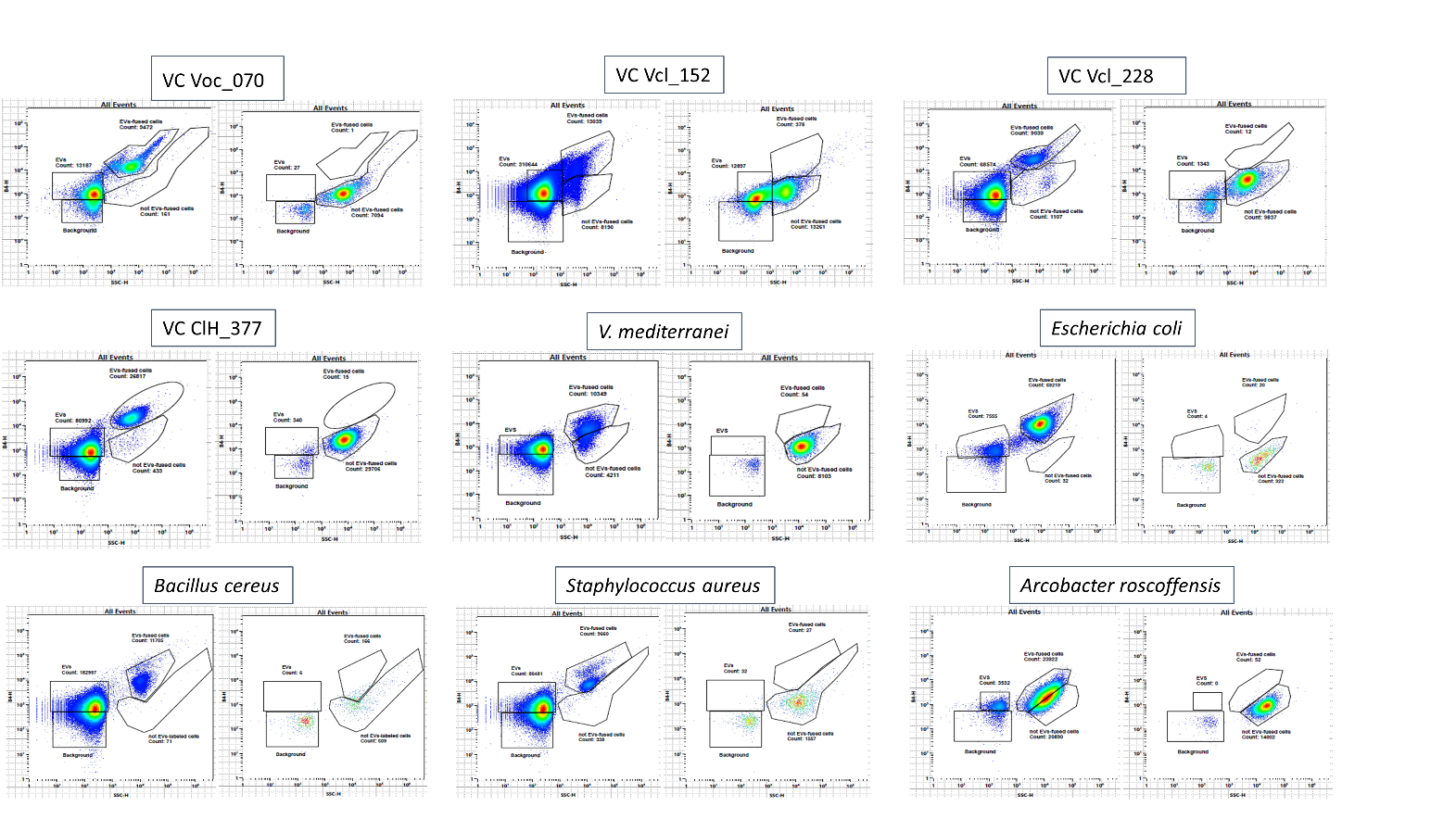
**

**Fig. S10**. Cytometry plots of fusion of *Vibrio coralliilyticus* strain Vic-Oc-068 EVs labeled with Alexa Fluor 488 and FM1-43, with other bacterial species (VC indicates *Vibrio coralliilyticus*). The left plot of each pair represents the sample (EVs+cells) while those on the right correspond to the negative control (HEPES + cells). *Vibrio coralliilyticus* strain Vic-Oc-068 EVs showed different interactions with different cells. Fusion, indicated by a shift in the cell population compared with the negative control, was observed with various *Vibrio coralliilyticus* strains, as well as with outliers such as *Escherichia coli* and Gram-positive bacteria including *Bacillus cereus* and *Staphylococcus aureus*. Not all cells in a culture fused with EVs, for instance, in *Arcobacter roscoffensis* and *Vibrio coralliilyticus* strain Vcl_152 cultures only a 53% or 57% of the cells fused with EVs. Cytometry plots shown are representative of the technical replicates. *Arcobacter roscoffensis, Escherichia coli* and VC ClH_377 plots shown do not have ~9000 events in the EVs-fused cells gate.


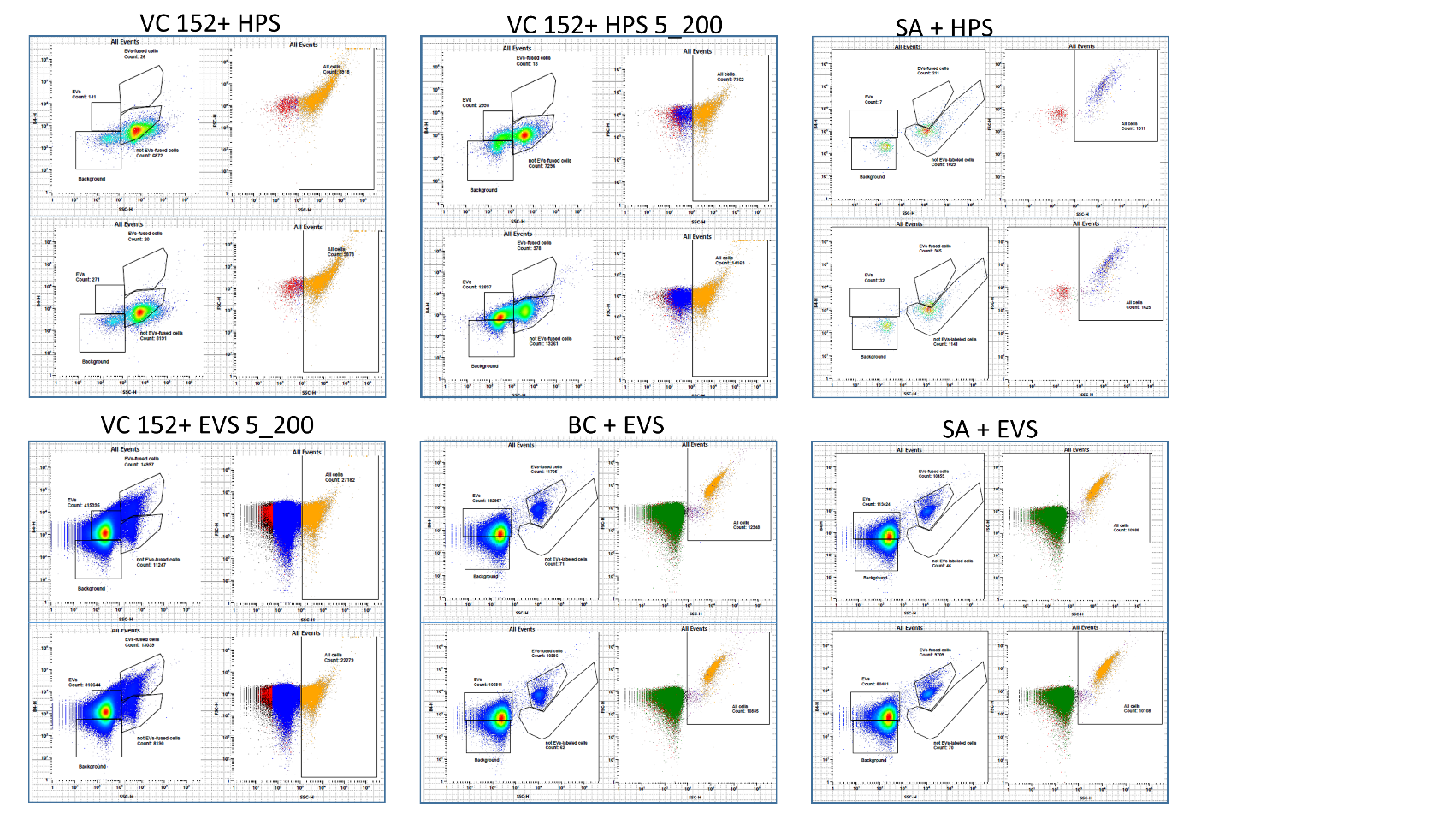


**Fig. S11.** Cytometer plots of technical duplicates of EVs-fusion experiments. Left panel plots fluorescence (BH4) vs SSCH, while right represents all cells gate (FSCH vs SSCH). Technical duplicates are inside the same square. Those technical replicates were performed to ensure the consistency in our measurements. Another technical replicate performed is showed in the Figure S7.


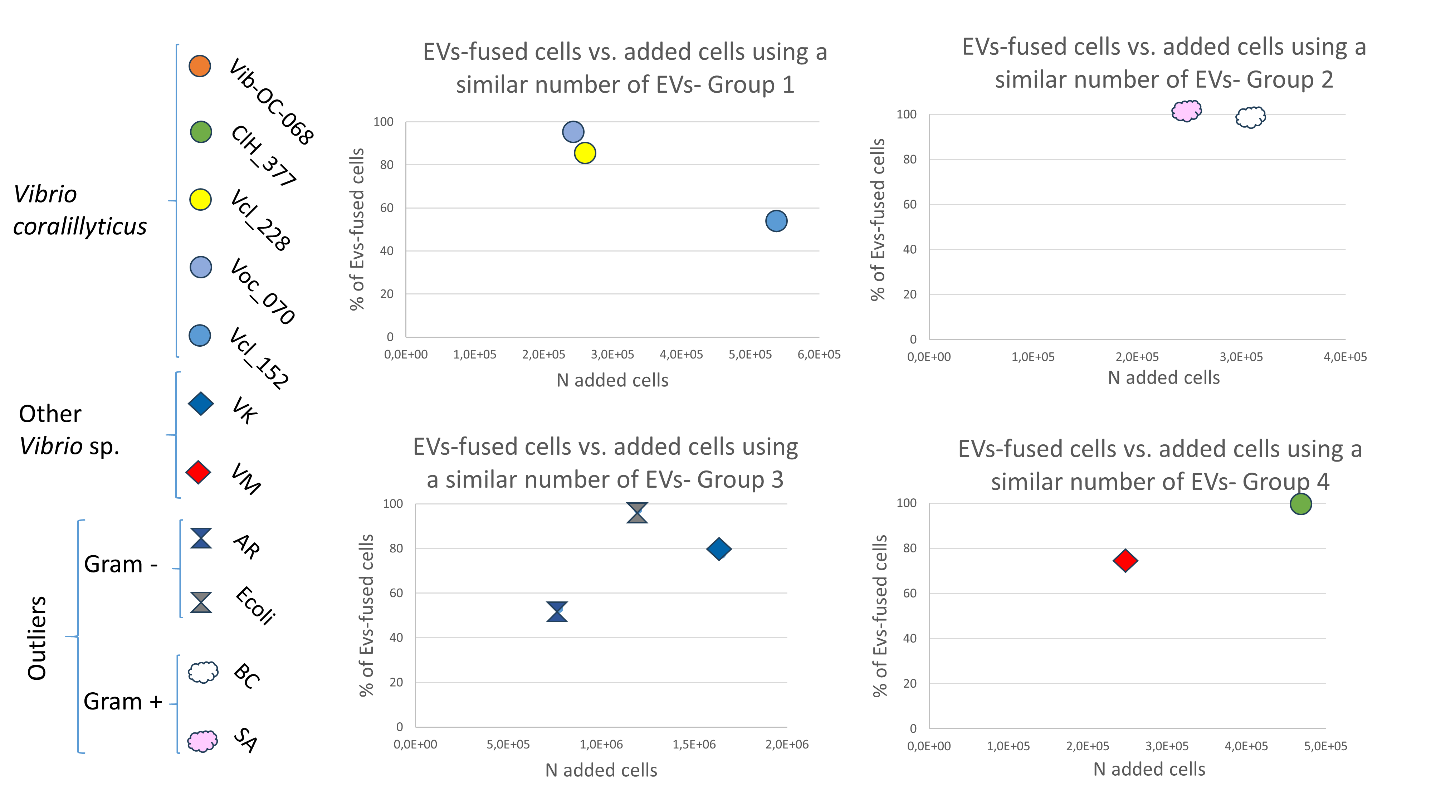


**Fig. S12.** For the fusion experiments, EVs of *Vibrio coralliilyticus* strain Vic-Oc-068 were incubated with various bacterial strains, which were grouped and exposed to the same amount of EVs as follows: Group 1 included *Vibrio coralliilyticus* (VC) strains Vcl_228, Voc_070 and Vcl_152; Group 2 included *Bacillus cereus* (BC) and *Staphylococcus aureus* (SA); Group 3 included *V. kanaloae* (VK), *Arcobacter roscoffensis* (AR) and *Esherichia coli*  *(*Ecoli); and Group 4 included *V. mediterranei* Vib-OC-097 (VM) and *Vibrio coralliilyticus* ClH_377*.* Experiments involving *Vibrio coralliilyticus* Vic-Oc-068 and its own EVs were conducted independently and are not represented in the graphs. These data are presented as descriptive evidence of consistent fusion/association patterns across recipient taxa rather than as a fully replicated inferential comparison.

***
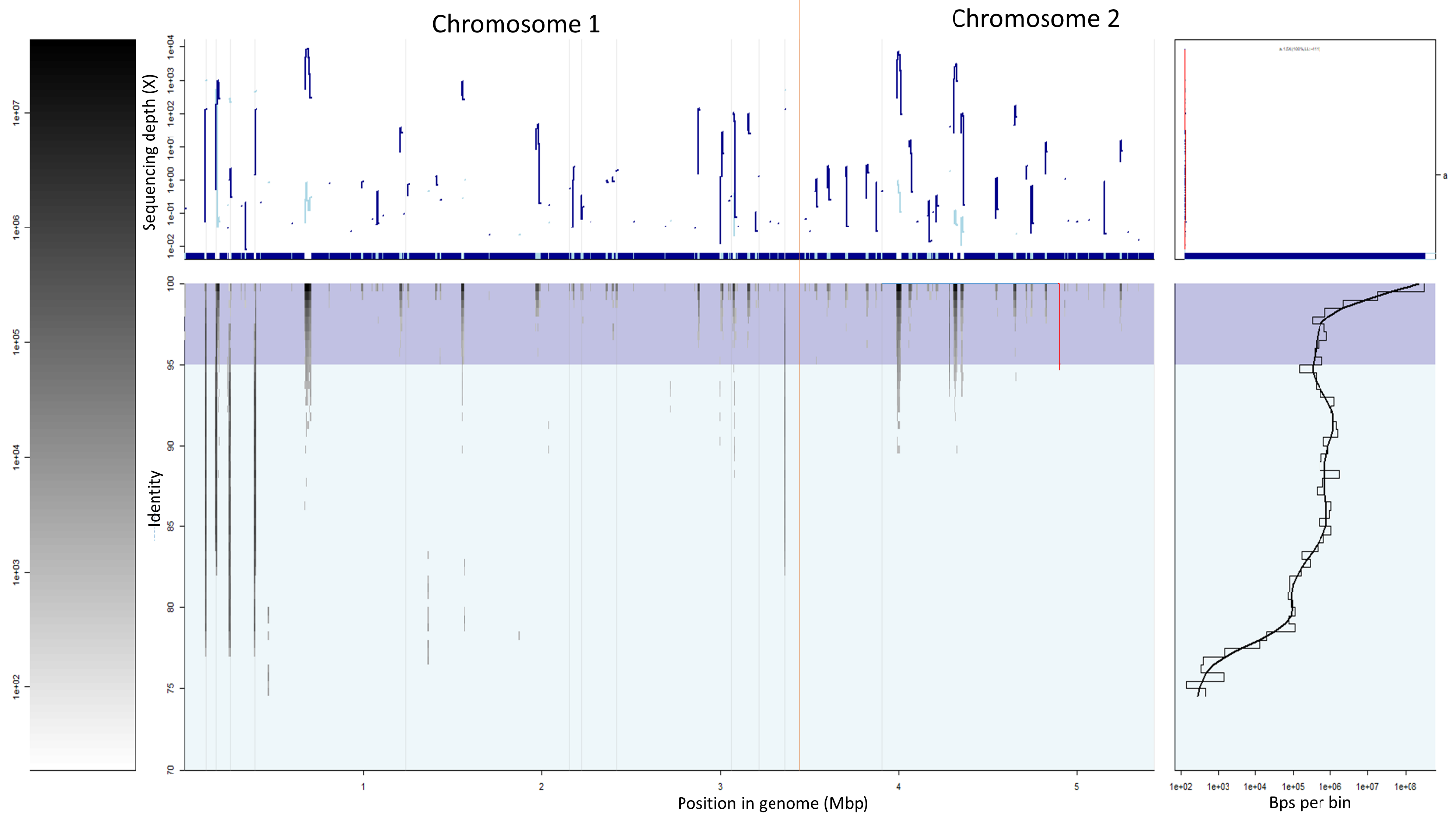
***

**Fig. S13** Mapping of EVS reads from *Vibrio coralliilyticus* strain Vic-Oc-068 to its own contig. DNA from the EVS of V. *coralliilyticus* strain Vic-Oc-068 was extracted and amplified using Multiple Displacement Amplification (MDA) to prepare a sequencing library. After a bioinformatic quality filtration, the reads were mapped to the bacterial contig. Only 15% of the reads mapped, showing an uneven distribution.

***Table S1.*** Bacterial strains used in this study.

| **Species and strain** | **Reference or source** |
| --- | --- |
| ***Vibrio coralliilyticus* Vib-OC-068 CECT 30097** | [2] |
| ***Vibrio coralliilyticus* ClH_377** | [3] |
| ***Vibrio coralliilyticus* Vcl_228** | [4] |
| ***Vibrio coralliilyticus* Voc_070** | [4] |
| ***Vibrio coralliilyticus* Vcl_152** | [4] |
| ***Vibrio kanaloae* strain 1C** | This work |
| ***Vibrio mediterranei* Vib-OC-097**  **CECT 30098** | [2] |
| ***Arcobacter roscoffensis*** | This work |
| ***Escherichia coli* CECT 101** | Spanish Type Culture Collection (Valencia, Spain) |
| ***Bacillus cereus* CECT 193** | Spanish Type Culture Collection (Valencia, Spain) |
| ***Staphylococcus aureus* CECT 435** | Spanish Type Culture Collection (Valencia, Spain) |

***Table S2.*** Increase in mean fluorescence for EVs-fused cell gates comparing sample and control.

|  | EVs *V. kanaloae* | EVs *V. coralliilyticus* Vic-Oc-068 |
| --- | --- | --- |
| cells *V. kanaloae* 1C | 1.95 | 2.50 |
| cells *V. coralliilyticus* Vic-Oc-068 | 2.56 | 1.71 |

***Table S3.*** DNA concentrations for 20% EVs fraction in LAB dil 1/5 and dialysis tubing cellulose membrane *V. kanaloae strain 1C* cultures.

| Sample name | DNA concentration (ng/μL) |
| --- | --- |
| EVs 20%_LAB dil 1/5 cultures | 5.29±0.66 |
| EVs 20%_Aquarium dil 1/5 cultures | 2.98±0.92 |

***Table S4.*** Sequencing data obtained from 20% EVs fraction isolated from dialysis tubing cellulose membrane culture of *V. kanaloae* strain 1C.

**Sample ID Total bases (bp) Total reads**

EVs_20%_Replicate_1 24,866,695,100 164,680,100

EVs_20%_Replicate_3 37,653,919,304 249,363,704

***Table S5.*** Quality filtering metrics for 20% EVs fraction purified from aquarium cultures *V. kanaloae* strain 1C.

| Sample name | Number of Reads | Total Number of Bases | Mean Read Length |
| --- | --- | --- | --- |
| EVs_20%1C_rep1 | 4,324,342 | 619,977,795 | 143.37 |
| EVs_20%1C_rep2 | 100,994,496 | 14,456,446,841 | 143.14 |

***Table S6.*** Quality filtering data of *Vibrio kanaloae strain 1C* HiFi PacBio long reads.

| Quality filtering data | Total reads (M) | Total bases (G) | Q20 bases (G) | Q30 bases (G) | GC content |
| --- | --- | --- | --- | --- | --- |
| Before quality filtering | 1.138.798 | 6.300.709 | 6.247.714 (99.15%) | 6.173.741 (97.98%) | 43.93% |
| After quality  filtering | 1.138.798 | 6.300.709 | 6.247.714  (99.15%) | 6.173.741 (97.98%) | 43.93% |

***Table S7.*** Genome assembly data of *Vibrio kanaloae* strain 1C using Flye assembler.

| Sample name | Number of contigs | Genome size (bp) | N50 (bp) |
| --- | --- | --- | --- |
| *Vibrio strain 1C* | 20 | 4.863.182 | 631.385 |

***Table S8.*** geNomad [5] scores for contigs with low mapping coverage.

| Seq_name | Chromosome_score | Plasmid_score | Virus_score |
| --- | --- | --- | --- |
| Concatenated_sequences_-_contig_29_(concatenated_sequence_13)-1cr3-correct | 0.0048 | 0.9827 | 0.0124 |
| Concatenated_sequences_-_contig_29_(concatenated_sequence_13)-1cr1 | 0.0048 | 0.9827 | 0.0124 |

***Table S9.*** Quality of sequenced data of *V.* *coralliilyticus* strain Vic-Oc-068 EVS which was MDA amplified.

| Quality filtering data | Total reads | Total bases (bp) | Q20 bases (%) | Q30 bases (%) | GC content  (%) |
| --- | --- | --- | --- | --- | --- |
| Before quality filtering | 23,653,224 | 3,571,636,824 | 97.3 | 92.6 | 58.7 |

***Table S10***. Kaiju classification of reads from *V.* *coralliilyticus* strain Vic-Oc-068 EVS that did not map to the contig of *V.* *coralliilyticus* strain Vic-Oc-068.

| Classification | % of total reads |
| --- | --- |
| *Vibrio* sp. (other than *V.* *coralliilyticus)* | 0.02 |
| *Achromobacter xylosoxidans* | 31.01 |
| Unclassified | 63.55 |
| Other bacteria sp. | 5.42 |

**References:**

1. Nekrouf NA, Maestre-carballa L, Lluesma-gomez M, Martinez-hernandez F, Martinez-garcia M. Annual dynamics and metagenomics of marine vesicles : One more layer of complexity in the dissolved organic fraction. 2025; 1–14.

2. Rubio-Portillo E, Yarza P, Peñalver C, Ramos-Esplá AA, Antón J. New insights into Oculina patagonica coral diseases and their associated Vibrio spp. communities. *ISME J 2014 89* 2014; **8**: 1794–1807.

3. Rubio-Portillo E, Izquierdo-Muñoz A, Gago JF, Rosselló-Mora R, Antón J, Ramos-Esplá AA. Effects of the 2015 heat wave on benthic invertebrates in the Tabarca Marine Protected Area (southeast Spain). *Mar Environ Res* 2016; **122**: 135–142.

4. Rubio-Portillo E, Gago JF, Martínez-García M, Vezzulli L, Rosselló-Móra R, Antón J, et al. Vibrio communities in scleractinian corals differ according to health status and geographic location in the Mediterranean Sea. *Syst Appl Microbiol* 2018; **41**: 131–138.

5. Camargo AP, Roux S, Schulz F, Babinski M, Xu Y, Hu B, et al. Identification of mobile genetic elements with geNomad. *Nat Biotechnol 2023 428* 2023; **42**: 1303–1312.
